# Deep Learning-Guided Discovery of Dual Inhibitors of SARS-CoV-2 Entry and 3CL Protease

**DOI:** 10.3390/molecules31061043

**Published:** 2026-03-20

**Authors:** Peng Gao, Ivan Pavlinov, Miao Xu, Catherine Z. Chen, Desarey Morales Vasquez, Qi Zhang, Yihong Ye, Luis Martinez-Sobrido, Wei Zheng, Min Shen

**Affiliations:** 1 Therapeutics Development Branch, Division of Preclinical Innovation, National Center for Translational Sciences (NCATS), National Institutes of Health (NIH), Rockville, MD 20850, USA; gaop3@nih.gov (P.G.); ivan.pavlinov@nih.gov (I.P.); miao.xu@nih.gov (M.X.); catherine.chen@nih.gov (C.Z.C.); qi.zhang3@nih.gov (Q.Z.); 2 Texas Biomedical Research Institute, San Antonio, TX 78227, USA; desarey.moralesvasquez@nationwidechildrens.org (D.M.V.); lmartinez@txbiomed.org (L.M.-S.); 3 National Institute of Diabetes and Digestive and Kidney Diseases (NIDDK), National Institutes of Health (NIH), Bethesda, MD 20892, USA; yihongy@niddk.nih.gov; 4 Early Translation Branch, Division of Preclinical Innovation, National Center for Translational Sciences (NCATS), National Institutes of Health (NIH), Rockville, MD 20850, USA

**Keywords:** SARS-CoV-2, 3CL protease, deep learning-based activity screening model, dual-target inhibition, heparan sulfate, graph convolutional networks, antiviral drug discovery

## Abstract

The rapid evolution of severe acute respiratory syndrome coronavirus 2 (SARS-CoV-2) underscores the need for antivirals that are resilient to resistance. Current Food and Drug Administration (FDA)-approved therapies primarily target single viral mechanisms, leaving gaps in efficacy. Here, we developed a Deep Learning-based Activity Screening Model (DLASM), which integrates graph convolutional network with machine learning to identify SARS-CoV-2 inhibitors, using experimental 3-chymotrypsin-like (3CL) main protease assay data. The optimized DLASMs virtually screened ~170,000 compounds from diverse in-house collections and yielded novel hits, several of which not only inhibited the 3CL protease but also blocked viral entry by interfering with heparan sulfate-mediated host interactions. These activities were validated through multiple assays, including 3CL enzymatic inhibition, SARS-CoV-2 pseudotyped particle entry, α-synuclein fibril uptake as a proxy for endocytosis, live virus cytopathic effect, heparan sulfate-dependent entry assay, and a 3D human lung mucociliary tissue model. Molecular docking studies elucidated binding modes at the 3CL protease active site, while molecular dynamics simulations provided insights into compound–heparan sulfate interactions. The identified compounds represent early-stage hits with moderate potency that demonstrate dual-mechanism antiviral activity. Together, these findings establish dual-target inhibition as a promising antiviral strategy, offering not only enhanced potency but also reduced risk of resistance. Moreover, our DLASM framework provides a generalizable pipeline for identifying chemically diverse scaffolds and for broader applications beyond SARS-CoV-2.

## 1. Introduction

SARS-CoV-2, a highly transmissible RNA virus, has caused unprecedented global health, social, and economic disruption since its emergence. Despite large-scale vaccination campaigns and the deployment of multiple antiviral therapies, COVID-19 continues to pose a major public health challenge due to ongoing viral evolution, immune escape, and the emergence of variants capable of causing breakthrough infections, even among vaccinated individuals [1,2,3,4,5]. Current therapeutic strategies primarily include vaccines, neutralizing antibodies, and small-molecule antivirals targeting viral replication. While these interventions have significantly reduced disease severity and mortality, their effectiveness can be limited by viral mutation, narrow target specificity, and the potential development of drug resistance. These limitations underscore the urgent need for next-generation antiviral approaches that are more resilient to viral evolution and capable of targeting multiple stages of the viral life cycle. SARS-CoV-2 transmission occurs mainly through inhalation of respiratory droplets from infected individuals. Viral entry into host cells is initiated by the interaction between the receptor-binding domain (RBD) of the viral Spike glycoprotein and the host cell-surface angiotensin-converting enzyme 2 (ACE2). In addition, heparan sulfate (HS), a negatively charged glycosaminoglycan, plays a critical role in facilitating and stabilizing viral attachment and entry into host cells [6,7]. We previously developed a deep learning classification model based on high-throughput screening data to identify inhibitors targeting HS proteoglycan and actin, highlighting the therapeutic potential of disrupting host-mediated viral entry mechanisms [8]. Following entry, viral replication proceeds through proteolytic cleavage of viral polyproteins encoded by open reading frames (ORFs) 1a and 1b, leading to the formation of the RNA replicase-transcriptase complex. Consequently, antiviral strategies that concurrently target both viral entry and replication may offer enhanced therapeutic efficacy and reduce the risk of resistance associated with SARS-CoV-2 evolution.

The 3CL protease (also known as 3CLpro or Mpro) is a key enzyme involved in the replication of coronaviruses, including SARS-CoV-2 [9,10]. This enzyme cleaves polyproteins translated from the viral RNA at specific sites into functional proteins, a prerequisite step for viral replication. Due to its critical role, the 3CL protease has become a primary target for antiviral drug development aimed at blocking viral replication. In the context of COVID-19, several drug candidates targeting the 3CL protease have been developed and evaluated as potential treatments. Notably, Nirmatrelvir, one of two components in Paxlovid, is a 3CL protease inhibitor that has been approved by FDA for the treatment of mild to moderate SARS-CoV-2 infections in adults at high risk of progressing to severe disease [11,12]. These drugs typically work by binding to the protease active site, inhibiting its function and thereby blocking viral replication. We recently reported the development of a fluorogenic 3CL protease enzymatic assay and the identification of early lead compounds through quantitative high throughput screening (qHTS) of ~5400 compounds, including approved drugs, clinically investigated drug candidates, and bioactive compounds [13,14].

In this study, we developed a double-layer screening framework called Deep Learning-based Activity Screening Model (DLASM), integrating both graph convolutional networks (GCN) and machine learning (ML) models for virtual screening. This combined approach offers an advantage over methods solely based on molecular graphs, as such methods rely on the extraction of 3D structural information, making prediction accuracy limited by the size and diversity coverage of training data [15,16,17,18]. Using 3CL qHTS experimental data from the National Center for Advancing Translational Sciences (NCATS)’ approved drug library, we built DLASMs and subsequently applied them to virtually screen approximately 170,000 compounds from various in-house chemical libraries. The 27 top-ranking hits that were confirmed by SARS-CoV-2 3CL enzymatic and pseudotyped particle entry assays were further evaluated by a battery of downstream assays and exhibited novel chemical scaffolds. Remarkably, several virtual screening hits not only inhibited 3CL protease activity but also blocked interactions with the cell-surface biopolymer heparan sulfate. We further validated these compounds using a plaque reduction neutralization test (PRNT) and in a human 3D mucociliary lung tissue model. Moreover, we conducted molecular docking studies to elucidate binding modes at the 3CL protease active site and performed molecular dynamics (MD) simulations to explore compound interactions with heparan sulfate. In summary, the double-layer computational framework provides a robust and generalizable virtual screening strategy for hit identification in early-stage antiviral drug discovery.

## 2. Results and Discussion

### 2.1. Overall Performance of the Double-Layer Deep Learning DLASM

Molecular graph-based approaches have demonstrated high performance in various structural modeling due to their ability to accurately extract structural information [15,16,17,18] while significantly reducing computational costs [19,20,21,22,23,24,25,26]. This architecture is well-suited for processing spatial information [27] and remains flexible enough to incorporate additional physicochemical properties to enhance classification performance [26,28,29,30,31,32,33,34,35,36,37]. However, solely relying on molecular graph-based methods may limit the ability to distinguish structurally similar compounds, particularly when the training set lacks sufficient molecular diversity. To address these limitations, we developed a double-layer architecture, the Deep Learning-based Activity Screening Model (DLASM), to enhance virtual screening efficiency. Utilizing the Deep Graph Library (DGL) [38,39], all the simplified molecular-input line-entry system (SMILES) strings of the collected compounds were converted into molecular graphs, where nodes represent atoms and edges denote inter-atomic connections. By encoding these features, the complete structural information is captured, with numerical values recorded in distance tensors at the radial basis function (RBF) layer during each evolution step to ensure no omission of atomic information. 

In order to identify candidate molecules targeting the 3CL protease, we first applied a self-developed classification model based on SchNet architecture for initial screening, leveraging the high-resolution of molecular graph representations [8,13]. Subsequently, a secondary ML-based screening layer was applied to further refine classification results, as illustrated in Section 3.1.

The SchNet component efficiently filters compounds based on structural similarity via extracting detailed spatial information from molecular graphs. As demonstrated by our previous study, SchNet can effectively exclude inactive compounds with high precision [8,40]. The integrated ML layer can further refine the virtual screening results from SchNet by incorporating numerical descriptors. For model generation, we developed the DLASM classification model using NCATS’ published experimental dataset, which was derived from a SARS-CoV-2 3CL protease enzymatic drug repurposing screen encompassing approximately 5400 compounds [13]. Data balancing and training/test sets splitting were described in the Experimental section. We integrated the SchNet classification module with four widely used ML algorithms: XGBoost, GBoost, RF, and SVM.

As shown in Table 1, the combination of SchNet with XGBoost consistently outperformed other machine learning methods across key performance metrics, achieving the highest AUC score (0.746) along with robust accuracy (0.81) and recall (0.51). These results suggest that SchNet&XGBoost not only distinguishes active from inactive compounds more effectively but also maintains a reasonable balance between precision and recall. To quantify the structural novelty of the identified hits, we calculated Tanimoto similarity coefficients between each of the 27 experimentally validated hits and all compounds in the training dataset using Morgan fingerprints (radius = 2, 2048 bits). The analysis revealed that 18 of the 27 hits (67%) exhibited maximum Tanimoto similarity < 0.4 to any compound in the training set, indicating substantial structural novelty. The median maximum Tanimoto similarity across all 27 hits was 0.35 (range: 0.22–0.68). Furthermore, Bemis–Murcko scaffold analysis showed that 22 of the 27 hits contained core scaffolds not represented in the training set. Together, these quantitative metrics indicate that the majority of identified actives occupy distinct chemical space underrepresented in the training data, demonstrating the model’s capability to generalize beyond the known chemotypes. Mechanistically, XGBoost contributes several practical advantages, including built-in regularization to mitigate overfitting, efficient processing of high-dimensional and sparse molecular descriptors, and the capacity to model complex non-linear relationships via gradient-boosted decision trees. These features make the SchNet&XGBoost framework particularly well suited for screening chemically diverse compound libraries and support its utility as a generalizable virtual screening approach.

### 2.2. Identification and Experimental Validation of 3CL Protease Inhibitors

**Figure 1:**
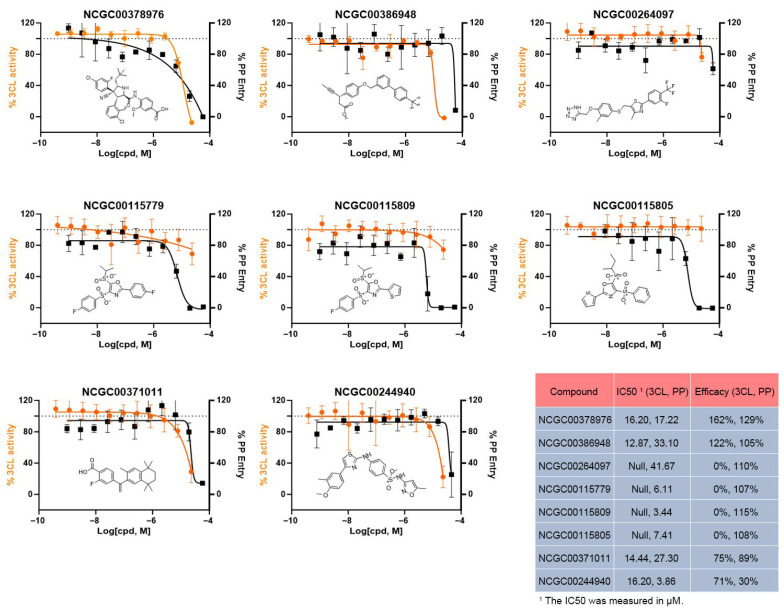
Experimental validation of the virtual screening hits in the 3CL protease assay and SARS-CoV-2 PP entry assay.

We applied the SchNet&XGBoost DLASM to screen ~170,000 compounds in the NCATS in-house collection. The virtual screening shortlisted around 500 top-ranked compounds, resulting in a manageable hits list for cherry-picking and experimental evaluation. These compounds were initially tested for activity in an in vitro 3CL protease enzymatic assay at a single concentration of 30 µM, identifying 70 compounds with >15% inhibition. Subsequently, these 70 compounds were further evaluated using an 11-concentration-point dose–response assay in triplicate for the 3CL enzymatic assay, as well as a SARS-CoV-2 pseudotyped particle (PP) cell entry assay, to confirm their activity against 3CL and assess their ability to block viral entry. Among these, 27 compounds exhibited reproducible activity in both the 3CL and PP assays. The resulting hit rate of 5.4% from the 500 virtual screening hits represents a 3.6-fold enrichment compared to the hit rate of 1.5% obtained in our previous drug repurposing screen. Examples of hits are shown in Figure 1.

Eight compounds were selected based on their potency, efficacy, structural novelty and differential activity profiles in both the 3CL enzymatic assay and the SARS-CoV-2 PP assay. NCGC00378976, NCGC00386948, NCGC00371011, and NCGC00244940 showed inhibition of 3CL protease with IC50 values ranging from 12.87 to 16.20 µM, indicating moderate enzymatic inhibition. Although these potencies reflect early-stage hit compounds that will require medicinal chemistry optimization, the compounds showed reproducible activity and modest inhibition in the PP viral entry assay, suggesting a potential dual mechanism involving both viral entry and replication. The remaining 4 compounds displayed activity only in PP viral entry assay, with little or no inhibitory effect on 3CL protease. Of particular interest, three of them (NCGC00115779, NCGC00115809, and NCGC00115805) shared the same chemical scaffold, disulfone-oxazole, indicating a possible common mechanism of action related to viral entry inhibition. Consistent with fingerprint analysis, comparison of their chemical structures with the training set confirms that these hits are structurally novel and distinct from previously reported molecules. We note that the observed 3CLpro inhibitory activity is relatively weak and primarily detectable at higher concentrations. Importantly, our study is focused on the discovery of non-covalent inhibitors, and these findings highlight the potential for identifying compounds that engage the target through reversible, non-covalent interactions rather than covalent modification. While the possibility of assay artifacts, such as compound aggregation or protein destabilization, cannot be fully excluded, the consistent activity across multiple orthogonal assays (3CL enzymatic, PP entry, cytopathic effect (CPE), plaque reduction neutralization test (PRNT), and EpiAirway) together with emerging structure–activity relationships support classification of these molecules as putative 3CLpro inhibitors. Future studies will incorporate additional biophysical validation methods, such as surface plasmon resonance and isothermal titration calorimetry, to further confirm direct and specific binding to 3CLpro.

**Figure 2:**
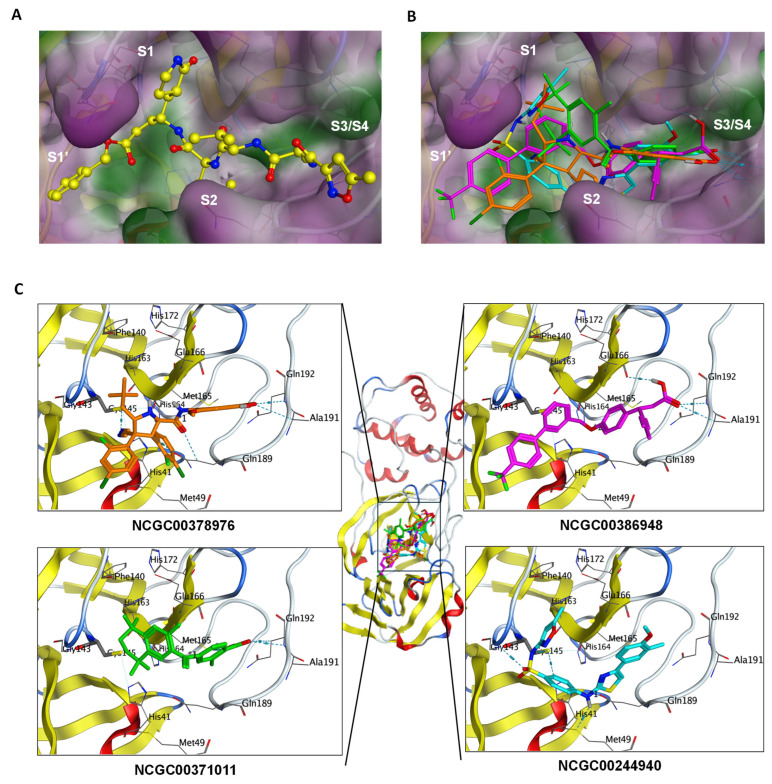
(**A**) Binding mode of the co-crystal ligand N3 in the 3CL protease active site. (**B**) Overlay of docking models for the four identified 3CL inhibitors within the active site. The protein surface is shown with hydrophobicity rendering (green = hydrophobic; purple = hydrophilic). (**C**) Detailed interaction modes of the docking models. Docked molecules are shown in stick representation, and hydrogen bonds are highlighted as blue dotted lines.

To unravel the possible binding mechanism of the identified 3CL protease inhibitors and guide future lead optimization, molecular docking was performed on NCGC00378976, NCGC00386948, NCGC00371011, and NCGC00244940 using the 3CL protease co-crystal structure. Molecular docking using the Molecular Operating Environment (MOE) platform yielded GBVI/WSA dG binding scores within a narrow range of −6.5 to −7.3 kcal/mol for NCGC00378976, NCGC00386948, NCGC00371011, and NCGC00244940, suggesting comparable predicted binding affinities under the same docking protocol. These scores are consistent with the observed micromolar IC_50_ values and reflect the non-covalent, reversible binding mode of these compounds. We note that the co-crystallized ligand N3 is a covalent inhibitor with a distinct binding mechanism not directly comparable to our non-covalent hits; nevertheless, the docking results support the ability of our compounds to establish favorable interactions within the 3CL protease active site through multiple hydrogen bonds and hydrophobic contacts. As depicted in Figure 2A,B, the binding mode of the reference co-crystal ligand N3 (PDB code: 6LU7, shown in yellow) is in comparison with the predicted docking models of those four lead compounds. As shown in Figure 2C, the tri-methyl group of NCGC00378976 inserts into the S1 pocket, which is composed of residues His163, Phe140, Glu166, His172 and Met165, while the other terminal phenyl-acid occupies the S3/S4 pocket. The 2,4-dichloro-phenyl and 2,3-dichloro-phenyl groups in the middle of the structure fit into the S1’ and S2 pockets, respectively, with the 2,3-dichloro-phenyl also forming a pi-stacking interaction with His41. In the NCGC00386948 docking model, the trifluoro-methylene group is positioned in the S1’ pocket, and the carboxylic acid at the other terminal fits into the S3/S4 pocket, forming H-bond interactions with Gln192 and Glu166. Similarly, for NCGC00371011, there is also a carboxylic acid moiety in the molecule that forms H-bond interaction with Glu192, indicating a critical role for this residue in 3CL inhibition. The dimethyl group of the cyclohexane ring fits into the S1 pocket. On the other hand, the methyl-oxazole moiety of NCGC00244940 occupies the S1 pocket, with the other terminal group fitting in the S3/S4 pocket. The sulfonamide oxygen forms an H-bond interaction with Gly143’s backbone -NH group, and another -NH linker forms an H-bond with Met49 near the S2 pocket. Collectively, these docking results suggest that the inhibitors bind near the 3CL protease active site through distinct binding modes, providing structural basis for future rational design of optimized inhibitors.

### 2.3. Prioritization of Hits by SARS-CoV-2 CPE Assay

**Figure 3:**
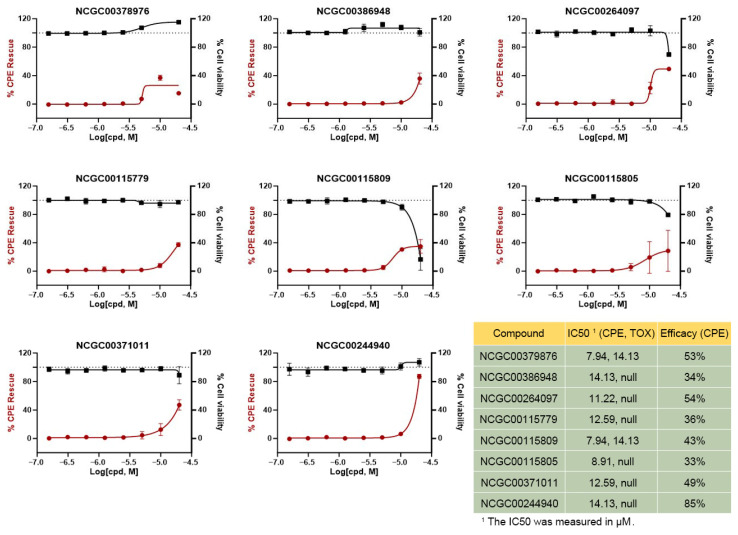
Experimental validation of the virtual screening hits in the SARS-CoV-2 CPE assay and Cytotox (TOX) assay. For compounds not achieving complete inhibition, relative IC_50_ values are reported as the concentration producing 50% of the maximal observed effect.

To verify the biological inhibition of the selected compounds against live SARS-CoV-2 virus, the cytopathic effect (CPE) assay was conducted [41]. The CPE assay is a relatively rapid and cost-effective method for screening compounds with antiviral activity. Promising candidates identified by this assay can guide compound prioritization for more comprehensive studies. In this assay, cells were infected with authentic SARS-CoV-2 virus, and the resulting cytopathic effects were measured via ATP content. These changes indicate the progression of viral infection and replication within the cells. In parallel, we also tested the cytotoxic effect of compounds in the same cells without virus infection. As depicted in Figure 3, all eight representative hits selected from 3CL protease assay and PP assay displayed varying degrees of activity in the CPE assay, with IC_50_ values ranging from 7.94 to 14.13 µM. It should be noted that, except for NCGC00244940, the tested compounds did not reach full inhibition of the cytopathic effect, and thus the reported IC_50_ values represent the concentration at which half of the maximal observed activity is achieved, rather than absolute half-maximal inhibition. The potency of these compounds will need to be further optimized through medicinal chemistry. The majority of the hits did not show cytotoxicity, except for NCGC00378976 and NCGC00115809, which showed mild cytotoxicity to host cells only at the highest tested concentration.

### 2.4. Mechanism of SARS-CoV-2 Viral Entry

**Figure 4:**
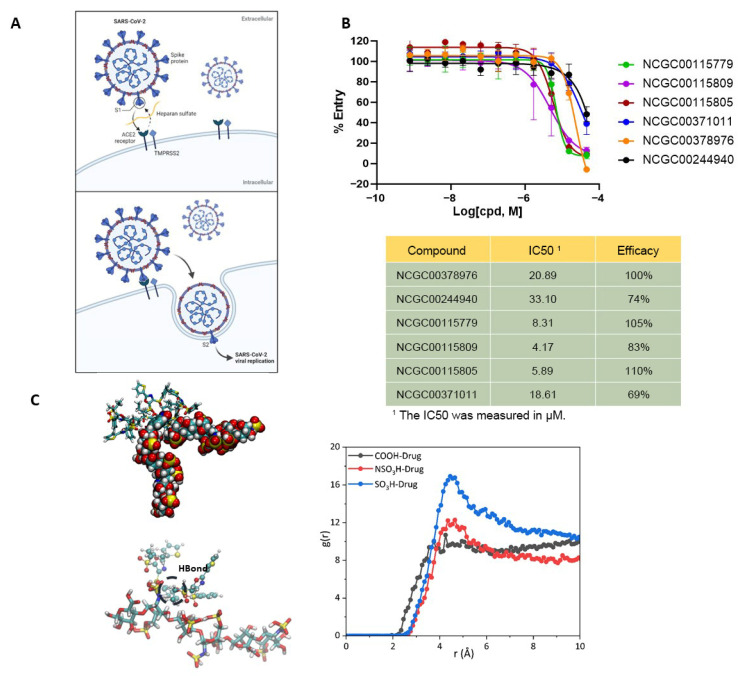
(**A**) Schematic illustration of the endocytosis-mediated viral entry mechanism. (**B**) Experimental validation of the selected compounds using the α-synuclein fibril uptake assay, showing inhibition of HS-mediated cellular uptake. (**C**) MD simulation of the interaction between the representative hit NCGC00115805 and HS. RDFs between NCGC00115805 and hydrogen bonding groups of HS are plotted to highlight key interaction sites.

After investigating the compounds’ inhibition of viral replication and cell entry, we explored their potential mechanism of action in mediating cell entry for these identified compounds. Previous studies, including ours, have shown that heparan sulfate proteoglycan (HSPG) facilitates SARS-CoV-2 cell entry [6]. The charged HS biopolymers play a pivotal role in mediating the interaction between cell-surface ACE2 and the Spike protein in SARS-CoV-2 infection. They also mediate the attachment and endocytosis of many non-viral cargos that bear clustered positive charges (Figure 4A). Similarly, the cellular uptake of α-synuclein fibrils is also an HSPG-dependent process. Preformed α-synuclein fibrils were widely used as a model to study the cell-to-cell spreading of pathogenic α-synuclein aggregates linked to Parkinson’s disease [42]. Our previous study shows that preformed α-synuclein fibrils use clustered lysine residues to bind to the cell surface HS, which facilitates their endocytosis. This process can be targeted by inhibitors that bind HS, which, as expected, also inhibits the entry of SARS-CoV-2 [6]. To assess the involvement of HSPG in SARS-CoV-2 cell entry for the 27 compounds that showed entry inhibition, we employed the α-synuclein fibrils uptake assay. We treated HEK293T cells with 200 nM α-synuclein fibrils labeled with red fluorescence together with the inhibitors identified from SARS-CoV-2 PP entry assay. As shown in Figure 4B, several compounds inhibited α-synuclein fibril uptake, with IC_50_ values ranging from 4.17 to 33.10 µM. These findings suggest that their mechanism of action may involve HS biopolymers or other cell surface molecules associated with endocytosis.

To further explore the underlying mechanism, MD simulations were conducted to examine the potential interactions between a representative hit (NCGC00115805) in complex with the HS biopolymer. As illustrated in Figure 4C, simulations of NCGC00115805 in complex with a 6-mer HS oligosaccharide revealed a tendency for the compound to preferentially localize near the –SO_3_H groups of HS, forming transient but recurrent hydrogen-bonding and electrostatic interactions between electron-donating and electron-accepting moieties. Throughout the 100 ns simulation, NCGC00115805 exhibited dynamic clustering around the sulfate groups despite the intrinsic conformational flexibility of the HS chain. Radial distribution function (RDF) analysis further supported this observation, showing preferential localization of the compound within 5 Å of –SO_3_H groups (see Supplementary Materials) and highlighting the –N–SO_3_H functionality as a major contributor to these interactions. Although the HS backbone displayed elevated root-mean-square deviation (RMSD) values (4–5 Å), this behavior is consistent with the known flexibility of HS oligosaccharides rather than ligand dissociation, as indicated by sustained hydrogen-bond occupancy (>60%) and relatively stable intermolecular distances during the simulation. These observations are consistent with previous reports [43,44], suggesting that compound-induced clustering or shielding of HS sulfate groups may disrupt the ability of HS to act as a co-receptor bridging the Spike protein and ACE2, thereby impairing viral entry. More broadly, these results suggest that interference with HS-mediated interactions may represent a generalizable host-targeting strategy, as HS proteoglycans are implicated in the entry mechanisms of multiple viruses. Understanding how small molecules modulate interactions between ubiquitous cell-surface glycans and viral proteins may therefore inform the development of broad-spectrum antiviral agents beyond SARS-CoV-2.

### 2.5. Confirmation of SARS-CoV-2 Inhibition by PRNT and EpiAirway Assays

**Figure 5:**
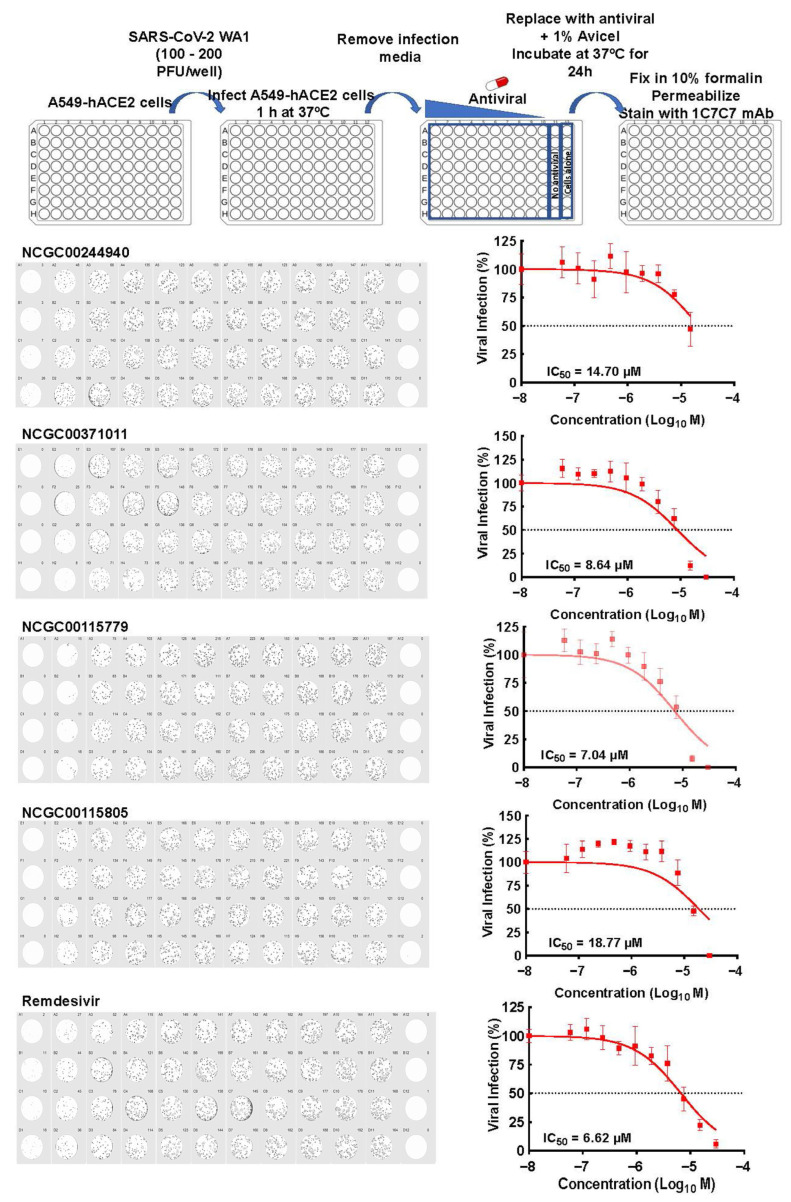
Workflow of the PRNT assay and results for the selected compounds compared with Remdesivir, demonstrating their inhibitory effects on SARS-CoV-2 plaque formation in A549-hACE2 cells.

We investigated four top-ranking hits that showed dual activity against 3CL and viral entry in more physiologically relevant SARS-CoV-2 infection assays. To this end, we employed a plaque reduction neutralization test (PRNT) assay using A549 lung cancer cells stably expressing human ACE2 (hACE2) [45] (Figure 5). Cells were first infected with SARS-CoV-2 WA1 and incubated with or without inhibitors for 24 h, followed by fixation and immunostaining. The plaque formation was then detected using the anti-SARS-CoV-2 nucleocapsid 1C7C7 monoclonal antibody. In this assay, all four lead compounds demonstrated inhibitory effects, with NCGC00371011 (IC_50_ = 8.64 µM) and NCGC00115779 (IC_50_ = 7.04 µM) exhibiting potencies comparable to Remdesivir (IC_50_ = 6.62 µM), which was included as a positive control and tested concurrently in our experiments. Remdesivir is an FDA approved SARS-CoV-2 replication inhibitor (Figure 5).

**Figure 6:**
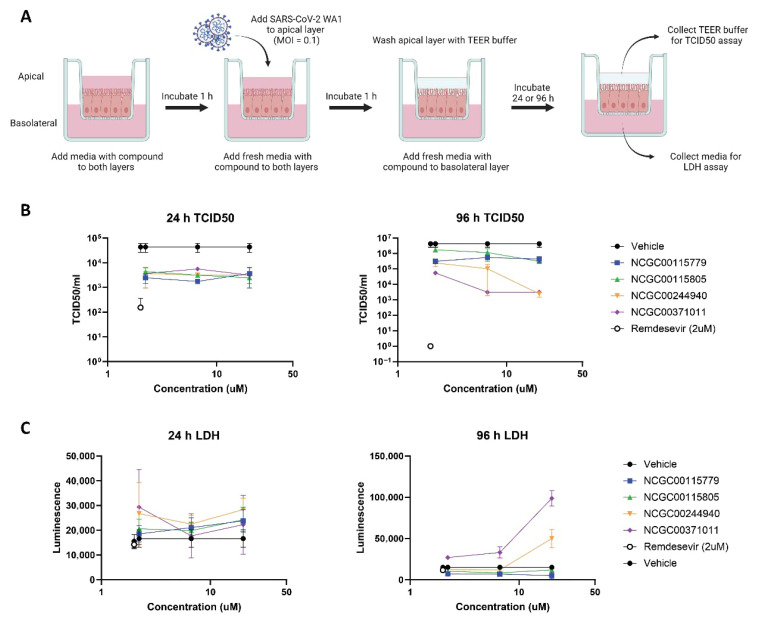
(**A**) Workflow of the EpiAirway 3D lung model assay. (**B**) TCID_50_ results in the EpiAirway assay after 24 h and 96 h of treatment with selected compounds (2.2, 6.6 and 20 µM) and Remdesivir (2 µM). (**C**) LDH assay results in the EpiAirway assay after 24 h and 96 h of treatment with selected compounds (2.2, 6.6 and 20 µM) and Remdesivir (2 µM).

To further evaluate our hits in a viral infection system that better captures the biology of the lung epithelium, we tested our molecules in the EpiAirway 3D lung model [46], in which primary human tracheal/bronchial epithelial cells are differentiated at an air-liquid interface to recreate the in vivo architecture and barrier function of the mucociliary epithelium (Figure 6A). All four compounds showed measurable efficacy, reducing the viral titers as measured by 50% tissue culture infectious dose (TCID_50_) at both 24 and 96 h, though not to the level of the Remdesivir control, which completely blocked viral replication. While all four compounds performed similarly at the 24 h timepoint, NCGC00244940 and NCGC00371011 out-performed the other two at the 96 h timepoint, reducing the viral titers by 100–1000 folds at the two higher concentrations (Figure 6B). However, both compounds also showed some cytotoxicity in the lactate dehydrogenase (LDH) assay at this timepoint, which may indicate that part of their antiviral effect may be attributable to infected-cell killing (Figure 6C). In contrast, NCGC00115779 and NCGC00115805 showed no cytotoxicity at 96 h, but only reduced viral titers by ~10-fold. Overall, NCGC00244940 emerged as the most promising candidate, reducing viral titers by 100-fold without noticeable cytotoxicity at 6.6 µM, which mirrors its activity in the CPE assay. Thus, its chemical scaffold appears suitable for future medicinal chemistry efforts to improve potency and efficacy.

## 3. Experimental Section

### 3.1. Double-Layer Deep Learning Architecture

The first layer consists of the SchNet module, a deep learning model originally designed for molecular and material property prediction, with the capability to perform classification tasks by learning atomic-level representations [38,47]. SchNet processes molecular structural data using continuous-filter convolutional layers to model quantum interactions and spatial arrangements. For classification, it predicts discrete class labels based on molecular features, such as chemical activity or toxicity. Its flexibility and high accuracy make it particularly well-suited for drug discovery and molecular design, supported by the PyTorch (v1.12)-based SchNetPack library. To further enhance the input representation, we integrated additional chemical descriptors from RDKit [48], including molecular weight, LogP (octanol-water partition coefficient), number of rotatable bonds, and topological polar surface area (TPSA). These descriptors provide complementary physicochemical information, enabling the model to leverage both learned atomic-level interactions and predefined molecular properties, leading to improved overall predictive performance in classification tasks.

**Figure 7:**
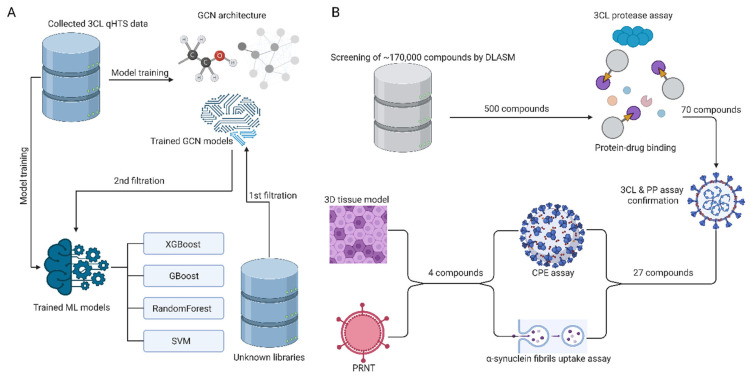
(**A**) Architecture of the DLASM integrating GCN molecular graph embeddings with ML classification. (**B**) Workflow of the virtual screening campaign and subsequent experimental validation leading to the identification of 3CL protease inhibitors.

The second layer employs machine learning (ML) models designed to refine and prioritize the initial candidates identified by SchNet. As shown in Figure 7, four classification algorithms were used: XGBoost (XGBClassifier) [49], Gradient Boosting (GradientBoostingClassifier) [50], Random Forest (RandomForestClassifier) [51], and Support Vector Machine (SVM) [52]. Model training and implementation were conducted in Python (v2.7) using the scikit-learn library with default parameters. Model performance was evaluated using the area under the receiver operating characteristic curve (AUC-ROC), which measures how well predicted values align with true labels in the test set, where a score of 1 indicates perfect classification and 0.5 reflects random performance in binary classification. To ensure robust evaluation, 3-fold cross-validation was performed with 10 random splits of the dataset, and AUC-ROC values were averaged.

### 3.2. Data Sets

The classification model was developed using NCATS’ published experimental dataset from the SARS-CoV-2 3CL protease enzymatic drug repurposing screen, which included approximately 5400 compounds [13]. Among these, 293 compounds demonstrated strong to moderate inhibition of 3CL protease and were categorized as actives. The activity classification was based on the following criteria: (1) compounds showing ≥50% inhibition at the highest tested concentration (typically 30–46 μM) in the 3CL protease enzymatic assay, AND (2) concentration-response curves fitting to curve class 1.1, 1.2, 2.1, or 2.2 according to NCATS curve class definitions, indicating reproducible concentration-dependent activity. Compounds showing <50% maximal inhibition or exhibiting inconsistent dose–response relationships (curve classes 3 or 4) were classified as inactive. To mitigate the effects of class imbalance while preserving chemical diversity, a diversity-aware under-sampling strategy was applied to the inactive compound set. Inactive compounds were first clustered using Tanimoto similarity calculated from ECFP4 fingerprints, with a similarity threshold of 0.6 to define structurally related groups. From each cluster, compounds were randomly selected in proportion to cluster size to ensure representative coverage of the inactive chemical space. Approximately 900 inactive compounds were selected, resulting in a final dataset of approximately 1200 compounds and maintaining an active-to-inactive ratio of roughly 1:3. Dataset splitting was performed using a stratified strategy, with 80% of the data used for training and 20% reserved for testing, while preserving the activity ratio in both subsets. This approach reduces bias toward the majority class while retaining representative chemical diversity, thereby improving model robustness and generalization. The trained SchNet model was evaluated for prediction accuracy and validated by the test set. Then it was applied to screen approximately 170,000 untested compounds from three NCATS in-house libraries: Sytravon, Genesis, and NCATS Pharmacologically Active Chemical Toolbox (NPACT). The Sytravon library is an in-house collection containing ~44,000 small molecules with chemically diverse and novel structures, as well as medicinal chemistry-tractable scaffolds. The Genesis library containing ~110,000 compounds was assembled to provide a novel modern chemical library that emphasizes high-quality chemical starting points, sp3-enriched chemotypes, and core scaffolds that enable rapid purchase and derivatization via medicinal chemistry. The NCATS Pharmacologically Active Chemical Toolbox (NPACT) is a collection of ~5000 annotated biologically active compounds. In total, more than 7000 mechanisms and phenotypes have been indicated in patents and published literature, with a wide coverage of biological interactions in microbial, mammalian, plant and other related systems. The evaluations of the physicochemical properties of these three libraries, as well as their structural diversity coverage can be found in our previous studies [8,40].

### 3.3. 3CL Protease Assay

The 3CL protease enzymatic assay was described in our previous study [53]. In a 384-well plate, 10 µL enzyme in reaction buffer was added into each well, and was incubated with a test compound for 30 min, followed by addition of 10 µL/well substrate. The fluorescent intensity was detected at different time points with the PHERAstar FSX plate reader (BMG Labtech, Cary, NC, USA) at Ex = 340 nm/Em = 460 nm after the substrate was added. The test was carried out at room temperature. Positive control (100% inhibition): reaction buffer with substrate only (no enzyme); negative control (0% inhibition): dimethyl sulfoxide (DMSO) vehicle (0.46% final concentration); reference inhibitor: GC376 (known 3CL inhibitor, tested in parallel). Data were normalized with wells containing enzyme and DMSO as 0% inhibition and wells containing substrate only as 100% inhibition.

### 3.4. SARS-CoV-2 Pseudotyped Particle (PP) Assay

The SARS-CoV-2 PP assay was conducted as previously described [54]. Human embryonic kidney (HEK) 293T-ACE2 cells seeded in white, solid bottom 384-well microplates (Greiner BioOne, Monroe, NC, USA) at 6000 cells/well in 15 µL medium were incubated at 37 °C with 5% CO_2_ overnight (~16 h). The drug compounds were titrated 1:3 with 11 points in DMSO and dispensed into the assay plate at 23 nL/well via pintool for testing. We incubated the cells with drug compounds for 1 h at 37 °C with 5% CO_2_ before 15 µL/well PPs were added. The plates were spinoculated by centrifugation at 1500 rpm (453× *g*) for 45 min and incubated for 48 h at 37 °C with 5% CO_2_ to allow PPs’ cell entry that expressing luciferase reporter. The supernatant was removed with gentle centrifugation by a Blue Washer (BlueCat Bio, Lebanon, NH, USA). Then 20 µL/well of Bright-Glo luciferase detection reagent (Promega, Madison, WI, USA) was added to the plates under room temperature with 5 min’ incubation. We measured the luminescence signal through the PHERAStar plate reader (BMG Labtech, Cary, NC, USA). Positive control (0% viral entry): HEK293T-ACE2 cells without PP addition; negative control (100% viral entry): HEK293T-ACE2 cells with PP and DMSO vehicle; reference inhibitor: Camostat mesylate (transmembrane serine protease 2 (TMPRSS2) inhibitor). Data were normalized with wells without no PP as 0% entry and wells containing PPs with DMSO as 100% entry.

### 3.5. SARS-CoV-2 Cytopathic Effect (CPE) Assay

The SARS-CoV-2 CPE test was carried out at Southern Research Institute (Birmingham, AL, USA) as reported in our previous studies [41]. Vero E6 cells expressing ACE2 were inoculated with SARS-CoV-2 (USAWA1/2020) at a multiplicity of infection (MOI) of 0.002 and treated with test compounds. After 72 h infection at 37 °C with 5% CO_2_, the cell viability was measured by the CellTiterGlo ATP content assay kit (Promega, Madison, WI, USA). Positive control (100% efficacy/no cytopathic effect): non-infected Vero E6-ACE2 cells; negative control (0% efficacy/maximum cytopathic effect): virus-infected cells with DMSO vehicle; reference antiviral: Remdesivir (tested in parallel). The CPE results obtained were normalized with respect to virus infected cells with DMSO as 0% efficacy and non-infected cells as 100% efficacy. For the cytotoxicity counter-screen, the cytotoxicity of the selected drug candidates was tested in the same cells by measuring the ATP content without virus infection. Negative control (100% viability): cells with DMSO vehicle (no compound, no virus); positive control (0% viability): media only (no cells). The obtained cytotoxicity results were normalized with wells containing media only as 0% viability (100% cytotoxicity) and wells containing cells with DMSO as 100% viability (0% cytotoxicity).

### 3.6. Heparan Sulfate Proteoglycan (HSPG) Dependent Endocytosis Assay

The fluorescence labeled α-synuclein fibrils used in this study were the same as reported in our previous study [10]. The HEK293T cells were dispensed into black, clear-bottom 1536-well microplates (Greiner BioOne, Monroe, NC, USA. # 789092-F) at 5000 cells/well in 5 μL medium with 200 nM pHrodo red-labeled α-synuclein fibrils and incubated overnight at 37 °C with 5% CO_2_ and 85% humidity (~16 h). The selected compounds were diluted at 1:3 ratio with 11 concentrations in DMSO and transferred to the assay plates at a volume of 23 nL/well by using an automated pintool workstation (Wako Automation, San Diego, CA, 91121, USA). After an incubation of 24 h, the fluorescence intensity of pHrodo red dye was measured in the CLARIOstar Plus plate reader (BMG Labtech, Cary, NC, USA). Positive control (100% internalization): HEK293T cells with 200 nM pHrodo red-labeled α-synuclein fibrils and DMSO vehicle; negative control (0% internalization): wells without cells; reference inhibitor: Surfen (known HS-binding compound). The results were normalized using wells without cells as 0% internalization, and wells with cells containing 200 nM pHrodo red-labeled α-synuclein fibrils and DMSO as 100% internalization, respectively.

### 3.7. Plaque Reduction Neutralization Test (PRNT) Assay

A549-hACE2 cells (96-plate format, 4 × 10^4^ cells/well, quadruplicates) were infected with SARS-CoV-2 USA/WA1/2020 (100–200 plaque-forming units (PFU)/well) for 1 h at 37 °C in a 5% CO_2_ atmosphere. After viral adsorption, the medium was aspirated and replaced with 100 µL of medium containing 10 concentrations of each compound at 1:2 dilution or 0.1% DMSO vehicle control with a final concentration of 1% Avicel (Sigma-Aldrich, St. Louis, MO 63103). Infected cells were incubated at 37 °C in a 5% CO_2_ atmosphere for 24 h. The cells were then fixed for immunostaining by removing medium and incubating with 10% neutral buffered formalin for 24 h at 4 °C. Cells were then washed with 1× phosphate-buffered saline (PBS) three times and permeabilized with 0.5% Triton X-100 in PBS for 15 min at room temperature. The plates were blocked with 2.5% bovine serum albumin (BSA) in PBS for 1 h at 37 °C, followed by incubation with 1 μg/mL of the SARS-CoV-2 anti-nucleocapsid (anti-N) monoclonal antibody (MAb) 1C7C7 for 1 h. Viral plaques were visualized using Vectastain ABC kit and 3,3′-diaminobenzidine (DAB) Peroxidase Substrate kit (Vector Laboratories Inc., Newark, CA, USA), following the manufacturer’s instructions. Stained plaques were scanned and analyzed using the Bioreader 7000-F-z-i plate reader and counting software (BIOSYS). Positive control: Remdesivir (tested at multiple concentrations in parallel); negative control: SARS-CoV-2 infected A549-hACE2 cells with 0.1% DMSO vehicle. The average and standard deviation (SD) of viral inhibition were calculated using Microsoft Excel software. Virus titers were calculated as plaque-forming units (PFU)/mL.

### 3.8. EpiAirway Assay

Infection in the 3D EpiAirway model was carried out by University of Louisiana as a contracted service and was previously described [55]. Human tracheobronchial epithelial cells (Epi-Airway™ from MatTek, Ashland, MA, USA) were cultured on inserts at an air-liquid interface in 6-well plates. Compounds were diluted in assay medium (AIR-100-ASY) and were added to each insert on the apical layer (0.15 mL) and basolateral layer (0.85 mL). After 1 h, the apical medium was removed, and the basolateral medium was replaced with fresh compound. Live SARS-CoV-2 USA/WA1/2020 virus (0.15 mL, MOI = 0.1) was then added to each insert on the apical layer, removed after 1 h, and washed with 0.4 mL of TEER buffer. The basal side medium/compound was replaced with 1 mL of assay medium. Every 24 h, the basolateral medium was replaced with 1 mL of fresh medium containing fresh compound. At 24 and 96 h postinfection (p.i.), the apical layer of the tissues was washed with 0.4 mL of trans-epithelial electrical resistance (TEER) buffer and aliquoted to separate microfuge tubes. At 24 and 96 h post-infection (p.i.), the basolateral medium (1.0 mL) was collected from each well, aliquoted into separate microfuge tubes, and stored at −80 °C. Apical layer supernatants from all treatments were titered by 50% tissue culture infectious dose (TCID_50_) to determine the amount of virus present in each sample. Medium from the basolateral layer collected at 24 and 96 h p.i. was assayed using a lactate dehydrogenase (LDH) release assay as a measure of cell viability. Positive control: Remdesivir (2 µM); vehicle control: assay medium without compound; mock infection control: uninfected tissues.

### 3.9. qHTS Data Analysis

The obtained quantitative high-throughput screening (qHTS) results were processed using a customized analysis client developed at NCATS. Raw data were first normalized by defining wells containing DMSO alone as 0% inhibition activity, and wells containing reaction buffer with substrate only (no enzyme) as 100% basal activity. To reduce experimental noise and minimize bias from single dose–response measurement, IC_50_ values were determined by global fitting of three independent concentration–response experiments, each performed in technical triplicate. Concentration–response curves and IC_50_ values were generated using GraphPad Prism software (v10.6) (San Diego, CA, 92108, USA). Standard deviations (SD) were calculated and included to represent variability across replicates.

### 3.10. Molecular Docking

The crystal structure of 3CL protease enzyme [56] (code: 6LU7) was obtained from Protein Data Bank (http://www.rcsb.org, accessed on 8 June 2023), at a resolution of 2.16 Å. The structure is complexed with a reported covalent inhibitor N3 within the active site [10]. Protein structure preparation work, including removing non-structural water molecules, hydrogen addition, protonation and conformational optimization was performed using QuickPrep module in Molecular Operating Environment (MOE v2020.09) software package (Chemical Computing Group ULC, Montreal, QC, Canada). Molecular docking was conducted using MOE package and guided by the co-crystal ligand N3 as the reference to define the docking site. For each molecule, thirty docking poses were generated using Triangle Matcher placement method and London dG scoring function, then followed by Rigid Receptor refinement for the top 5 poses using GBVI/WSA dG scoring function. The binding affinity values of the top candidates obtained from docking were used to estimate the strength of interaction between these compounds and the active sites of 3CL protease.

### 3.11. MD Simulation

The MD simulations were conducted using the GROMACS 2019.6 package [57], and the CHARMM27 force field was employed to investigate the interactions between the identified potent compounds and HS biopolymer [58,59,60]. To ensure the stability of the initial configuration, energy minimization was performed using the steepest descent method for the first 10,000 steps with a convergence criterion of 100 kJ/mol/nm. Then, the production simulations were run for 100 ns under the NPT ensemble, with a time step of 2.0 fs, for each compound-HS system. Three independent 100 ns MD simulations were performed for the NCGC00115805-HS system, starting from different initial coordinates and velocities. TIP3P water model was used for solvation [61,62], with a physiological saline concentration of 0.15 M. All simulations were conducted with periodic boundary conditions (PBCs), and bond constraints were processed using LINear Constraint Solver (LINCS) algorithm [63,64]. Long-range electrostatic interactions were calculated using the particle mesh Ewald (PME) method, with a cutoff of 1.4 nm [65]. The simulation temperature was maintained at 310 K using the V-rescale thermostat, and pressure was controlled at 1 bar using the Berendsen barostat.

## 4. Conclusions

Both viral entry and replication play crucial roles in viral infection and propagation. To effectively combat COVID-19, therapeutic candidates that simultaneously target both processes could potentially reduce susceptibility to the evolution of viral resistance. Molecular graph- and ML-based combinational screening approaches, such as DLASM, can serve as valuable complements to qHTS in drug discovery campaigns. In this study, by screening untested, large diverse chemical libraries, we successfully identified several novel candidate compounds with antiviral activity, validated through a battery of SARS-CoV-2 related assays. The identified compounds represent early-stage hits with moderate potency (IC_50_ values in the 4–16 µM range) that will require medicinal chemistry optimization to improve binding affinity and efficacy. Molecular docking analyses provided insight into plausible binding modes and key amino acid residues involved in interactions with the 3CL protease, offering a structural basis to guide future optimization efforts. Notably, several compounds exhibited dual inhibitory activity, targeting both the viral 3CL protease and host cell HSPG. Cell-based uptake assays and MD simulations suggest that certain compounds interfere with HSPG-mediated endocytosis by clustering around sulfate groups on HS, implicating cell-surface heparan sulfate as an additional host target. Together, these findings indicate a two-pronged mechanism of action involving inhibition of both viral and host factors, which may enhance antiviral efficacy and reduce the likelihood of resistance development. Overall, this work provides new chemical starting points for the development of dual-action antivirals targeting both viral entry and intracellular replication, and highlights the flexibility and utility of our screening strategy. By integrating multiple orthogonal biological assays with targeted AI-driven virtual screening models, this approach offers a broadly applicable framework for accelerating drug discovery beyond COVID-19 and addressing other pressing therapeutic challenges.

## Figures and Tables

**Table 1:** Performance metrics of SchNet combined with different machine learning algorithms.

Method ^(*a*)^	AUC Score	Precision	Accuracy	Test Recall
1. SchNet&SVM	0.661	0.43	0.72	0.55
2. SchNet&RF	0.692	0.64	0.80	0.36
3. SchNet&GBoost	0.732	0.71	0.83	0.47
4. SchNet&XGBoost	0.746	0.62	0.81	0.51

*^(a^*^)^ The technical details can be found at our GitHub page: https://github.com/tcsnfrank0177/3CL_VirtualScreening (accessed on 2 January 2026).

## Data Availability

The computational files and technique details can be found in our GitHub page: https://github.com/tcsnfrank0177/3CL_VirtualScreening (accessed on 2 January 2026).

## Supplementary Materials

The following supporting information can be downloaded at: https://www.mdpi.com/1420-3049/31/6/1043/s1. The QC data, root-mean-square deviations (RMSDs) for MD simulations and RDF analysis, binding activity analysis and the enzymatic and PP assay test results of the lead compounds can be found in Supporting Information. Table S1. Selected compounds upon 3CL protease assay validation; Table S2. Selected compounds upon PP assay validation; Table S3. Selected compounds upon α-Synuclein fibrils uptake assay validation; Table S4. Selected compounds upon CPE assay validation; Figure S1. The RMSDs of the MD simulations (including three replica copies starting from different coordinates and velocities) of the interaction between NCGC00115805 and HS; Figure S2. Radial distribution functions (RDFs) between NCGC00115805 and the hydrogen bonding functional groups of HS.
