# Supplementary material for: Deep Learning-Guided Discovery of Dual Inhibitors of SARS-CoV-2 Entry and 3CL Protease
Source: Molecules. 2026 Mar 20;31(6):1043. doi: 10.3390/molecules31061043 (PMC13319945; doi:10.3390/molecules31061043)

# Supporting Information

# Deep Learning-Guided Discovery of Dual Inhibitors of SARS-CoV-2 Entry and 3CL Protease

**Peng Gao [1], Ivan Pavlinov [1], Miao Xu [1], Catherine Z. Chen [1], Desarey Morales Vasquez [2], Qi Zhang [1], Yihong Ye [3], Luis Martinez-Sobrido [2], Wei Zheng [1,*] and Min Shen [4,*]**

[1] Therapeutics Development Branch, Division of Preclinical Innovation, National Center for Translational Sciences (NCATS), National Institutes of Health (NIH), Rockville, MD 20850, USA
[2] Texas Biomedical Research Institute, San Antonio, TX 78227, USA
[3] National Institute of Diabetes and Digestive and Kidney Diseases (NIDDK), National Institutes of Health (NIH), Bethesda, MD 20892, USA
[4] Early Translation Branch, Division of Preclinical Innovation, National Center for Translational Sciences (NCATS), National Institutes of Health (NIH), Rockville, MD 20850, USA

* Correspondence: wzheng@mail.nih.gov (W.Z.); shenmin@mail.nih.gov (M.S.)

Table S1. Selected compounds upon 3CL protease assay validation:

| Sample ID | IC50 (uM) | Efficacy | Max Resp |
|---|---|---|---|
| NCGC00119962-01 | 4.069652 | -30.635 | -14.374 |
| NCGC00426057-01 | 7.236978 | -49.403 | -18.08 |
| NCGC00105207-01 | 12.869369 | -45.313 | -18.925 |
| NCGC00141309-01 | 8.120023 | -49.61 | -28.467 |
| NCGC00119986-01 | 14.43967 | -60.153 | -16.708 |
| NCGC00119988-01 | 7.236978 | -44.383 | -25.117 |
| NCGC00386948-05 | 14.43967 | -135.02 | -93.69 |
| NCGC00119932-01 | 16.201576 | -34.816 | -1.551 |
| NCGC00101532-01 | 16.201576 | -71.83 | -17.041 |
| NCGC00124832-01 | null | -41.377 | -1.668 |
| NCGC00454367-01 | null | -26.06 | -0.232 |
| NCGC00457024-01 | null | -28.519 | -11.974 |
| NCGC00109048-01 | null | -22.735 | -0.916 |
| NCGC00112920-01 | null | -26.342 | -3.072 |
| NCGC00132939-01 | null | -25.829 | -7.584 |
| NCGC00120065-01 | null | -32.418 | -17.766 |
| NCGC00104296-01 | null | -16.665 | -3.166 |
| NCGC00109537-01 | null | -25.313 | 5.332 |
| NCGC00135082-01 | null | -40.082 | -17.195 |

Table S2. Selected compounds upon PP assay validation:

| Sample ID | IC50 (uM) | Efficacy | Max Resp |
|---|---|---|---|
| NCGC00025064-05 | 0.863288 | -103.994 | -99.382 |
| NCGC00386667-01 | 3.856164 | -107.5 | -100.368 |
| NCGC00110915-01 | 4.854623 | -114.759 | -98.845 |
| NCGC00015874-15 | 9.686246 | -98.909 | -100.14 |
| NCGC00100377-03 | 0.769406 | -114.447 | -97.254 |
| NCGC00115753-04 | 6.111608 | -107.662 | -99.001 |
| NCGC00379247-02 | 10.868146 | -134.991 | -90.84 |
| NCGC00115775-02 | 9.686246 | -97.566 | -100.331 |
| NCGC00115811-02 | 7.694058 | -105.824 | -98.432 |
| NCGC00871455-01 | 7.694058 | -102.556 | -98.424 |
| NCGC00115779-03 | 6.111608 | -106.961 | -98.417 |
| NCGC00115787-02 | 7.694058 | -126.099 | -98.373 |
| NCGC00117664-06 | 0.432669 | -95.489 | -99.625 |
| NCGC00110923-01 | 5.446976 | -122.574 | -99.286 |
| NCGC00411718-01 | 9.941799 | -127.366 | -98.372 |
| NCGC00104274-02 | 19.326601 | -128.651 | -100.346 |
| NCGC00244940-01 | 3.856164 | 38.165 | -30.06 |
| NCGC00264097-01 | null | 0 | -31.897 |
| NCGC00351745-02 | null | 0 | -78.451 |

Table S3. Selected compounds upon α-Synuclein fibrils uptake assay validation:

| Sample ID | AC50 (uM) | Efficacy | Max Resp |
|---|---|---|---|
| NCGC00025064-05 | 1.861476 | -83.773 | -86.554 |
| NCGC00386667-01 | 14.78623 | -113.827 | -86.526 |
| NCGC00110915-01 | 3.714133 | -90.335 | -31.493 |
| NCGC00015874-15 | 16.590423 | -102.368 | -64.662 |
| NCGC00100377-03 | 3.714133 | -103.547 | -92.772 |
| NCGC00115753-04 | 3.714133 | -91.667 | -86.337 |
| NCGC00379247-02 | 4.675817 | -135.712 | -90.676 |
| NCGC00115775-02 | 7.41067 | -115.21 | -87.777 |
| NCGC00115811-02 | 8.314908 | -106.352 | -80.661 |
| NCGC00115779-03 | 8.314908 | -104.786 | -88.089 |
| NCGC00115787-02 | 4.675817 | -86.505 | -87.822 |
| NCGC00117664-06 | 3.714133 | -125.954 | -101.939 |
| NCGC00110923-01 | 3.310225 | -84.741 | -68.302 |
| NCGC00411718-01 | 3.397559 | -110.288 | -81.267 |
| NCGC00104274-02 | 8.314908 | -94.621 | -61.033 |
| NCGC00351745-02 | 6.604766 | -81.481 | -64.767 |
| NCGC00871455-01 | 20.886106 | -70.987 | -57.264 |
| NCGC00264097-01 | null | 0 | -2.02 |

Table S4. Selected compounds upon CPE assay validation:

| Sample ID | AC50 (uM) | Efficacy | Max Resp |
|---|---|---|---|
| NCGC00264097-01 | 11.220185 | 53.591 | 49.73 |
| NCGC00115779-03 | 12.589254 | 36.343 | 39.339 |
| NCGC00104274-02 | 7.943282 | 102.826 | -1.664 |
| NCGC00411718-01 | 11.220185 | 58.256 | 14.192 |
| NCGC00015874-15 | 8.912509 | 83.761 | -1.835 |
| NCGC00386667-01 | 8.912509 | 37.424 | -2.434 |
| NCGC00871455-01 | 7.943282 | 132.135 | -2.148 |
| NCGC00115753-04 | null | 14.37 | 3.913 |
| NCGC00117664-06 | null | -15.927 | -2.409 |
| TRND00489059-01 | null | 21.5 | 20.966 |
| NCGC00115755-02 | null | 14.445 | -1.019 |
| NCGC00386266-01 | null | 12.463 | 13.178 |
| NCGC00135082-01 | null | 14.371 | 16.422 |
| NCGC00169844-02 | null | 8.276 | 2.495 |
| NCGC00264094-01 | null | 12.796 | 15.748 |
| NCGC00380658-01 | null | 5.185 | 5.176 |
| NCGC00100377-03 | null | -16.427 | -2.439 |
| NCGC00411727-01 | null | 13.852 | 17.354 |
| TRND00492315-01 | null | 24.519 | 36.271 |
| NCGC00015735-25 | null | 19 | 20.253 |
| NCGC00115775-02 | null | 17.016 | 16.944 |

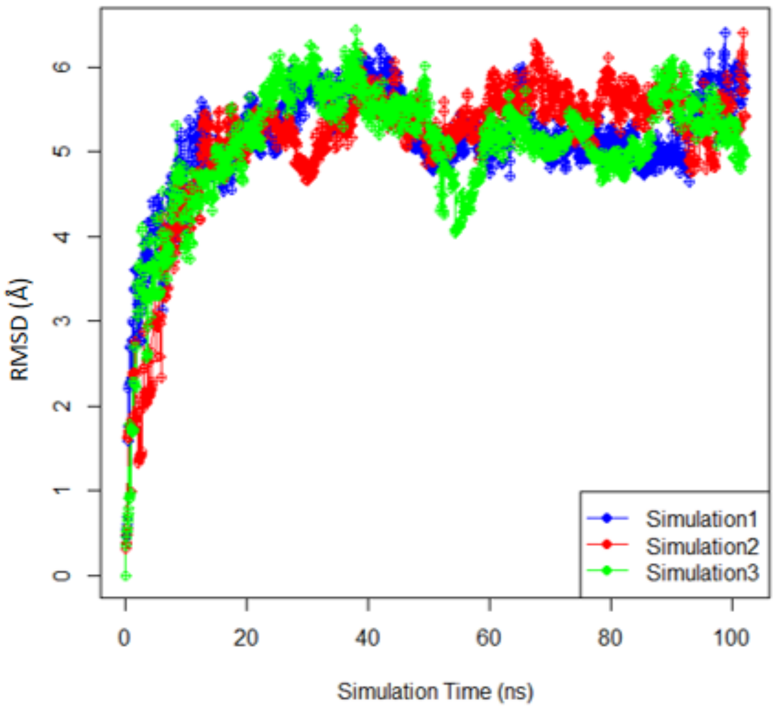


Figure S1. The RMSDs of the MD simulations (including three replica copies starting from different coordinates and velocities) of the interaction between NCGC00115805 and HS.

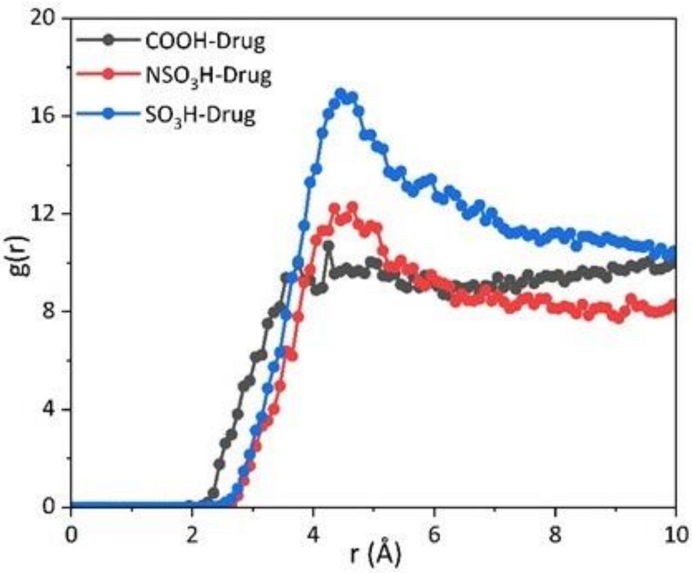


Figure S2. Radial distribution functions (RDFs) between NCGC00115805 and the hydrogen-bonding functional groups of HS. The RDF, *g(r)*, describes the probability of finding a pair of atoms at a distance r relative to an ideal gas distribution, thereby characterizing spatial correlations between interacting groups. Peaks in the RDF indicate preferred intermolecular distances and reveal key bond interaction sites between NCGC00115805 and HS.

QC analysis was performed on reported compounds to confirm identity, purity, and data integrity prior to downstream evaluation:

NCGC00115779

NCGC00115805

NCGC00115805

NCGC00244940

NCGC00264097

NCGC00371011

NCGC00378976

NCGC00386948

File ..mschem\03-23\310323-NCGC00115779-03-08131.D Tgt Mass (EZX): 427.04
Injection Date : 31-Mar-23, 15:52:55 Seq. Line : 0
Sample Name : NCGC00115779-03 Location : D1B-D6
Acq. Operator : Hsiu-Ling Lin Inj : 1
Spec. Reported : UV Integration Inj Volume : -3 ul
Acq. Method : C:\Users\Public\Documents\ChemStation\1\Methods\FINAL_GRAD_NO_PRINT.M
Analysis Method : C:\Users\Public\Documents\ChemStation\1\Methods\FINAL_GRAD_NO_PRINT.M
Sample Info : 0222273653 WalkUp method: 'FINAL_GRD_NO PRINT' Mol Wt: 427.04
Method Info : FINAL GRD BUT NO PRINT

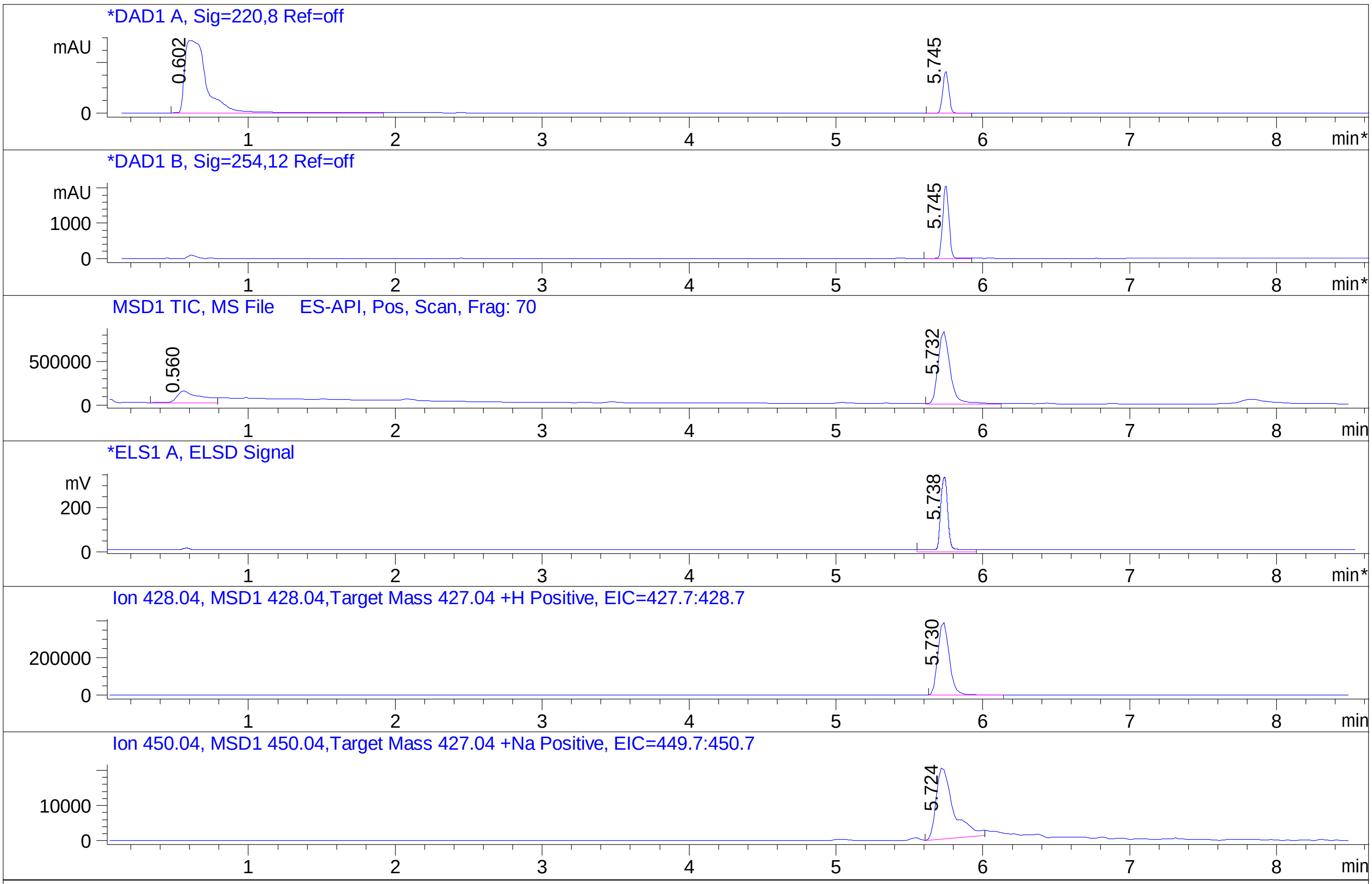


Integration Results for DAD1 A, Sig=220,8 Ref=off

| RetTim | Width | Area | Height | Area% | MS(+) |
|---|---|---|---|---|---|
| 0.60 | 0.16 | 30330.64 | 2887.73 | 86.48 | 179 |
| 5.74 | 0.05 | 4741.80 | 1663.06 | 13.52 | 428 |

Integration Results for DAD1 B, Sig=254,12 Ref=off

| RetTim | Width | Area | Height | Area% | MS(+) |
|---|---|---|---|---|---|
| 5.74 | 0.05 | 6022.91 | 2077.53 | 100.00 | 428 |

Ret. Time: 0.60 <<<< POSITIVE SPECTRA >>>> Ret. Time: 5.74

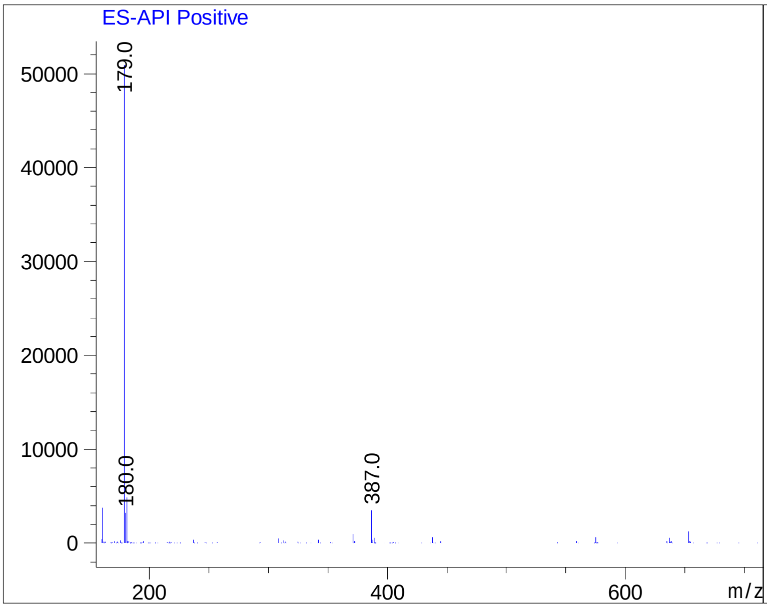


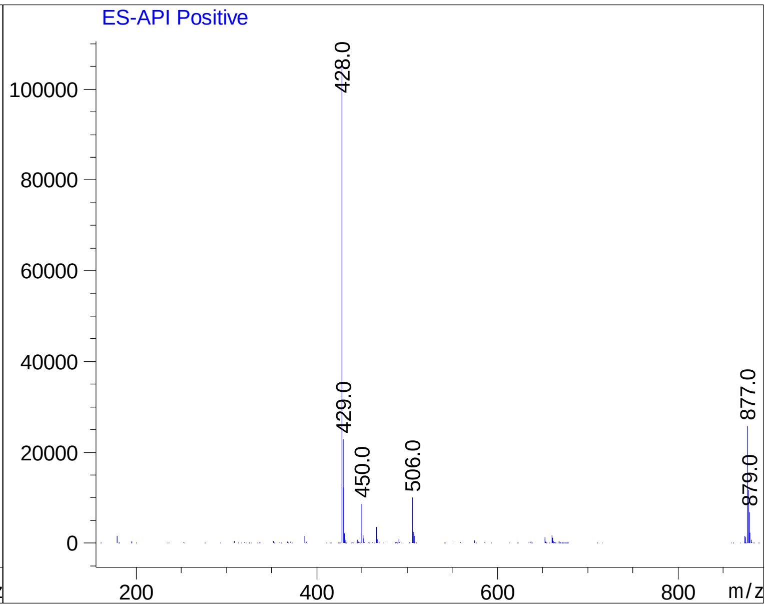

**File ..mschem\03-23\310323-NCGC00115805-02-08132.D Tgt Mass (EZX): 411.03**
**Injection Date : 31-Mar-23, 16:03:15 Seq. Line : 0**
**Sample Name : NCGC00115805-02 Location : D1B-D7**
**Acq. Operator : Hsiu-Ling Lin Inj : 1**
**Spec. Reported : UV Integration Inj Volume : -3 ul**
**Acq. Method : C:\Users\Public\Documents\ChemStation\1\Methods\FINAL_GRAD_NO_PRINT.M**
**Analysis Method : C:\Users\Public\Documents\ChemStation\1\Methods\FINAL_GRAD_NO_PRINT.M**
**Sample Info : 0222273605 WalkUp method: 'FINAL_GRD_NO PRINT' Mol Wt: 411.03**
**Method Info : FINAL GRD BUT NO PRINT**

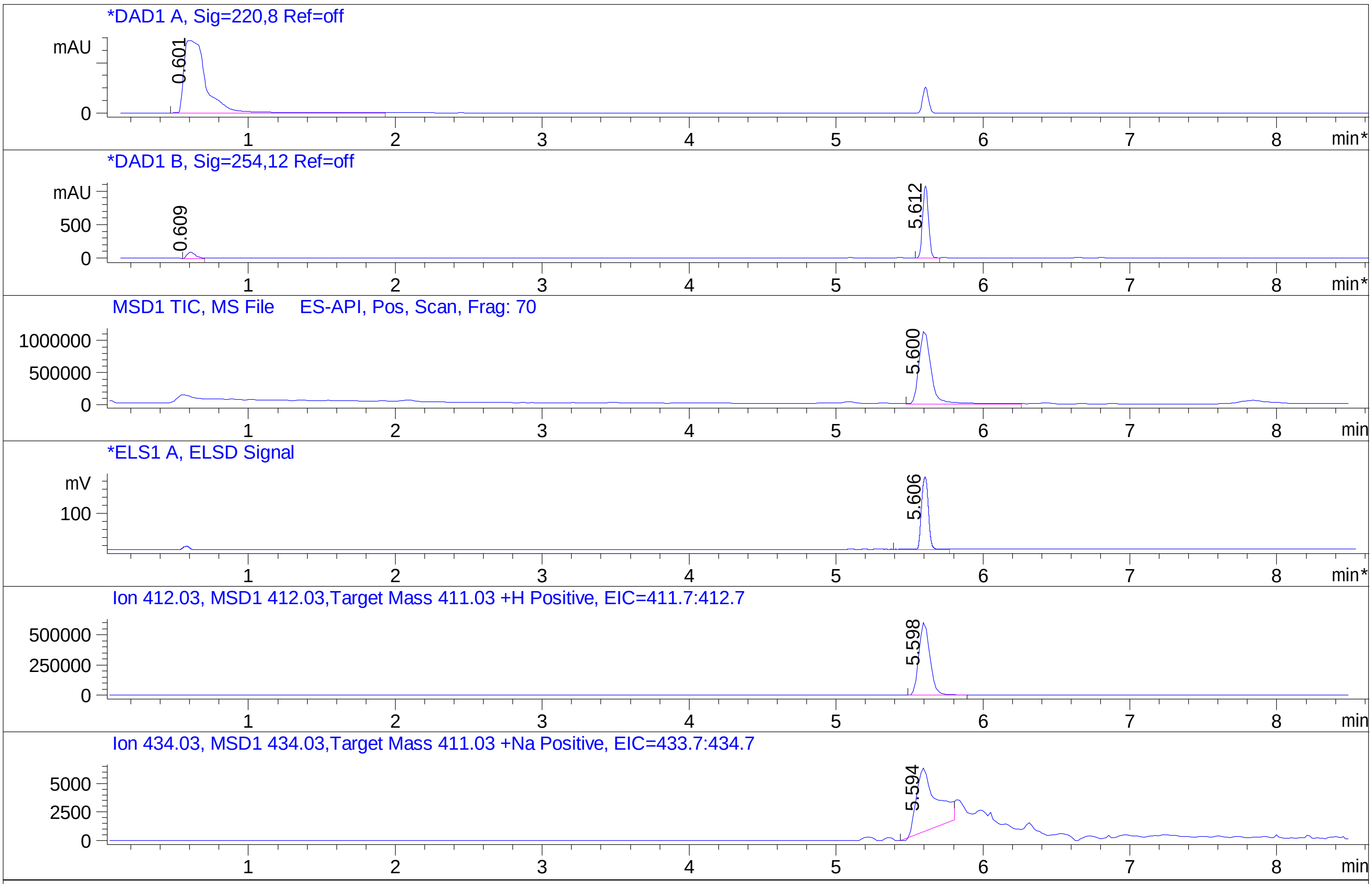


Integration Results for DAD1 A, Sig=220,8 Ref=off

| RetTim | Width | Area | Height | Area% | MS(+) |
|---|---|---|---|---|---|
| 0.60 | 0.16 | 30582.59 | 2894.23 | 100.00 | 179 |

Integration Results for DAD1 B, Sig=254,12 Ref=off

| RetTim | Width | Area | Height | Area% | MS(+) |
|---|---|---|---|---|---|
| 0.61 | 0.06 | 400.97 | 99.15 | 11.68 | 179 |
| 5.61 | 0.04 | 3032.84 | 1079.81 | 88.32 | 412 |

Ret. Time: 0.60  <<<< POSITIVE SPECTRA >>>>    Ret. Time: 5.61

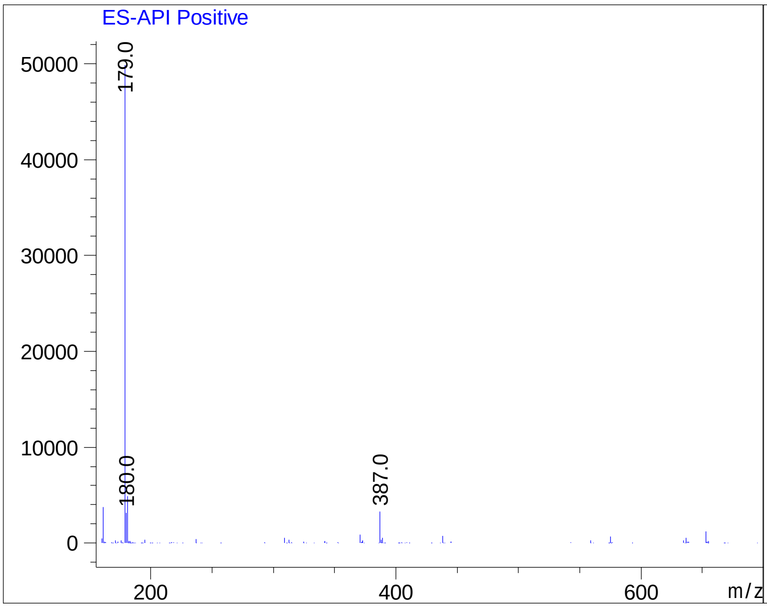


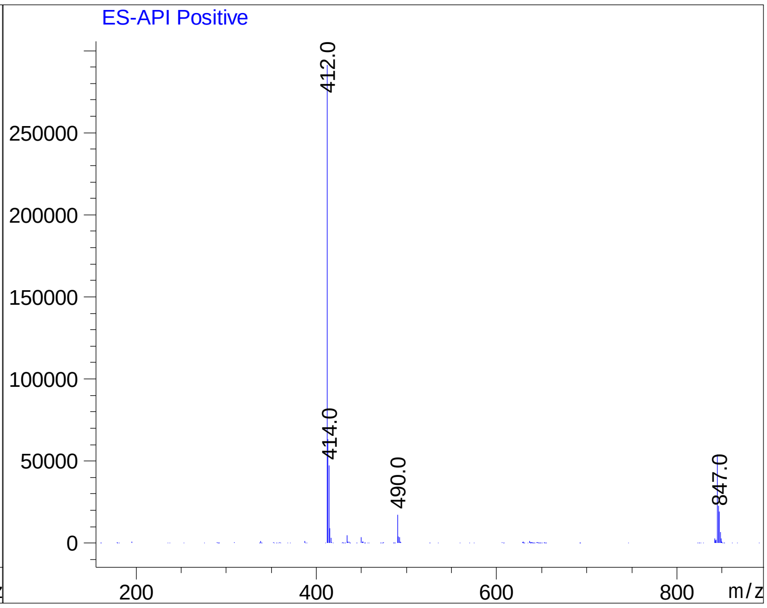

**File ..mschem\04-23\200423-NCGC00115809-03-08267.D Tgt Mass (EZX): 415.00**
**Injection Date : 20-Apr-23, 12:30:10 Seq. Line : 0**
**Sample Name : NCGC00115809-03 Location : D2F-D1**
**Acq. Operator : Hsiu-Ling Lin Inj : 1**
**Spec. Reported : UV Integration Inj Volume : -3 ul**
**Acq. Method : C:\Users\Public\Documents\ChemStation\1\Methods\FINAL_GRAD_NO_PRINT.M**
**Analysis Method : C:\Users\Public\Documents\ChemStation\1\Methods\FINAL_GRAD_NO_PRINT.M**
**Sample Info : 0222273584 WalkUp method: 'FINAL_GRD_NO PRINT' Mol Wt: 415.00**
**Method Info : FINAL GRD BUT NO PRINT**

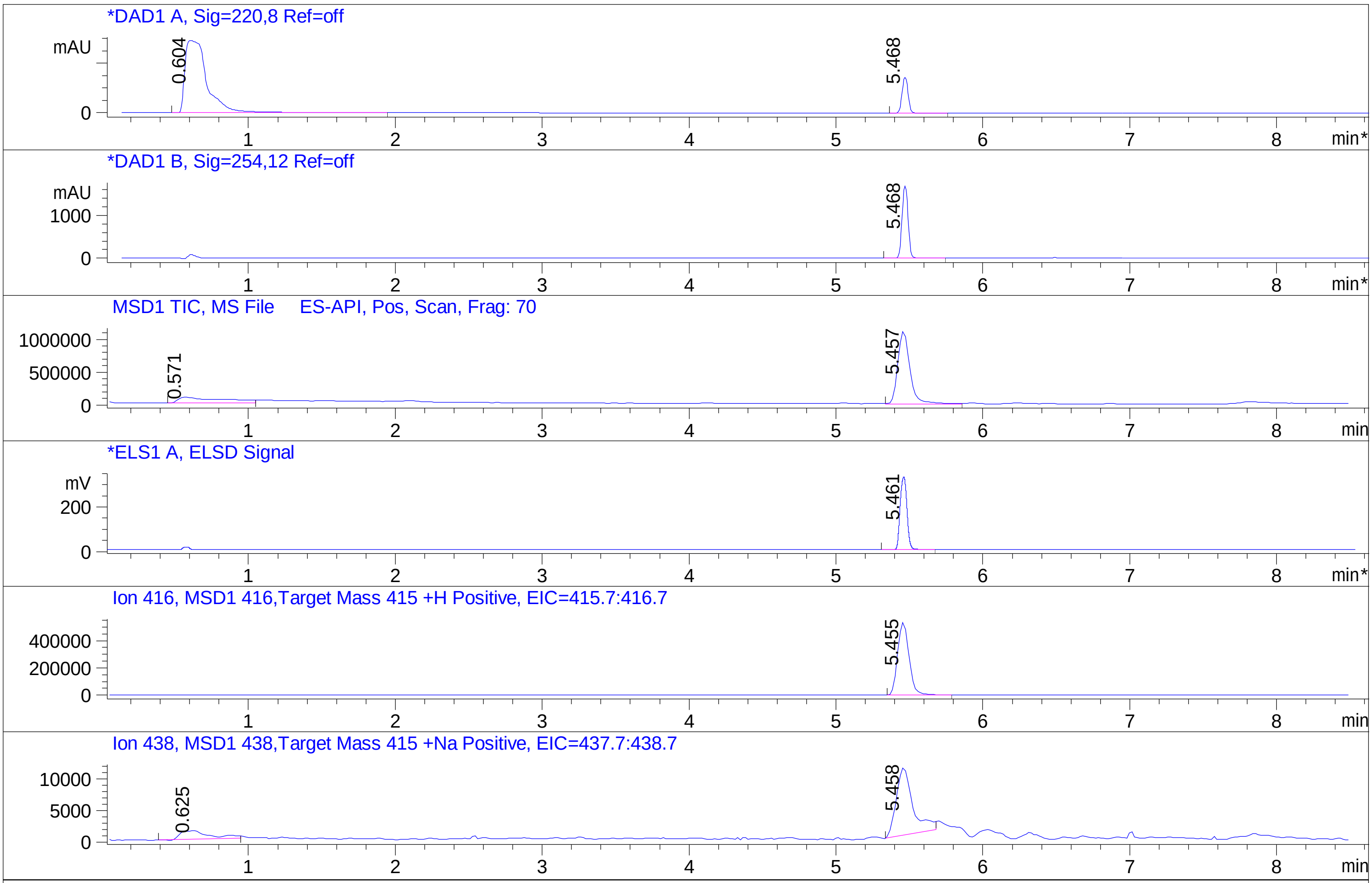


Integration Results for DAD1 A, Sig=220,8 Ref=off

| RetTim | Width | Area | Height | Area% | MS(+) |
|---|---|---|---|---|---|
| 0.60 | 0.14 | 30781.19 | 2917.47 | 88.23 | 179 |
| 5.47 | 0.05 | 4105.66 | 1450.19 | 11.77 | 416 |

Integration Results for DAD1 B, Sig=254,12 Ref=off

| RetTim | Width | Area | Height | Area% | MS(+) |
|---|---|---|---|---|---|
| 5.47 | 0.05 | 4800.17 | 1687.53 | 100.00 | 416 |

Ret. Time: 0.60 <<<< POSITIVE SPECTRA >>>> Ret. Time: 5.47

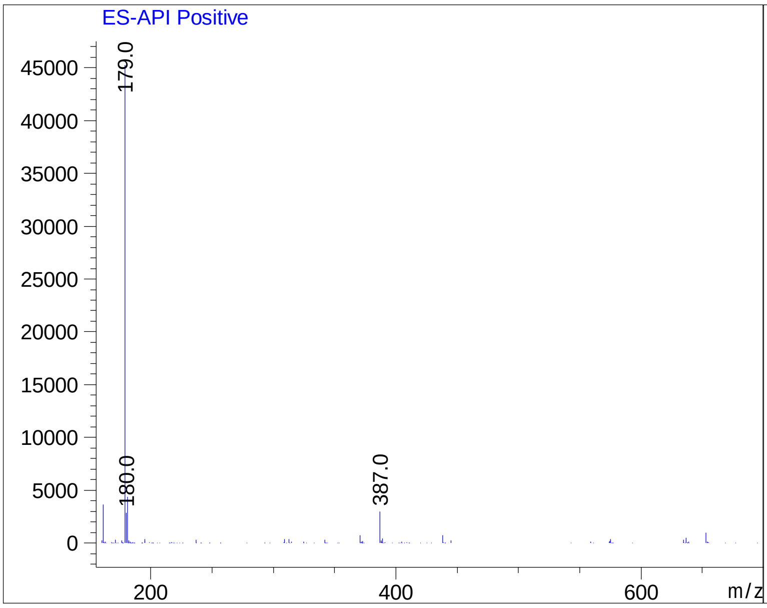


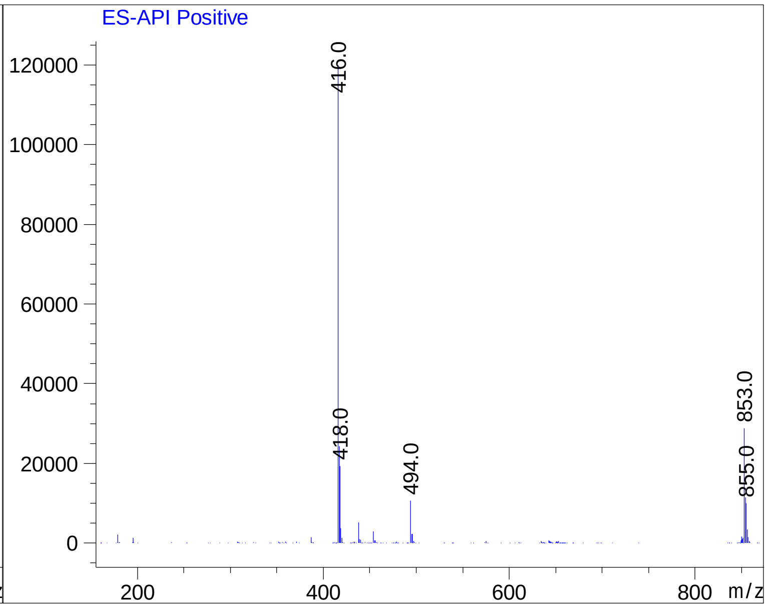

File ..mschem\08-21\170821-NCGC00244940-01-02768.D Tgt Mass (EZX): 456.09
Injection Date : 17-Aug-21, 14:07:44 Seq. Line : 0
Sample Name : NCGC00244940-01 Location : D2F-A1
Acq. Operator : Katlin Recabo Inj : 1
Spec. Reported : UV Integration Inj Volume : -3 ul
Acq. Method : C:\Users\Public\Documents\ChemStation\1\Methods\FINAL_GRAD_NO_PRINT.M
Analysis Method : C:\Users\Public\Documents\ChemStation\1\Methods\FINAL_GRAD_NO_PRINT.M
Sample Info : 0059781862 WalkUp method: 'FINAL_GRD_NO PRINT' Mol Wt: 456.09
Method Info : FINAL GRD BUT NO PRINT

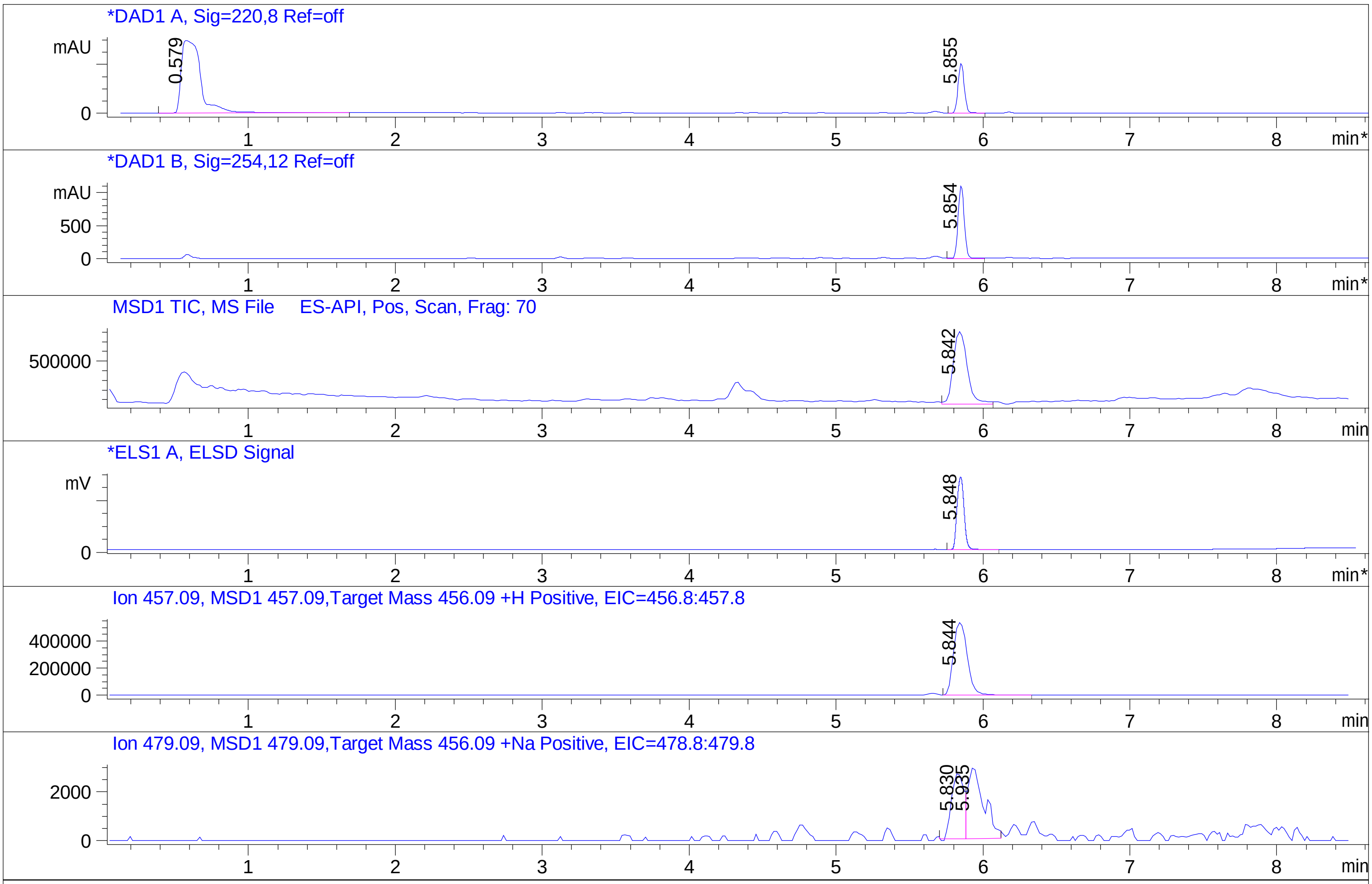


Integration Results for DAD1 A, Sig=220,8 Ref=off

| RetTim | Width | Area | Height | Area% | MS(+) |
|---|---|---|---|---|---|
| 0.58 | 0.15 | 27507.76 | 2990.16 | 81.92 | 179 |
| 5.85 | 0.05 | 6071.33 | 2042.32 | 18.08 | 457 |

Integration Results for DAD1 B, Sig=254,12 Ref=off

| RetTim | Width | Area | Height | Area% | MS(+) |
|---|---|---|---|---|---|
| 5.85 | 0.05 | 3243.83 | 1102.29 | 100.00 | 457 |

Ret. Time: 0.58 <<<< POSITIVE SPECTRA >>>> Ret. Time: 5.85

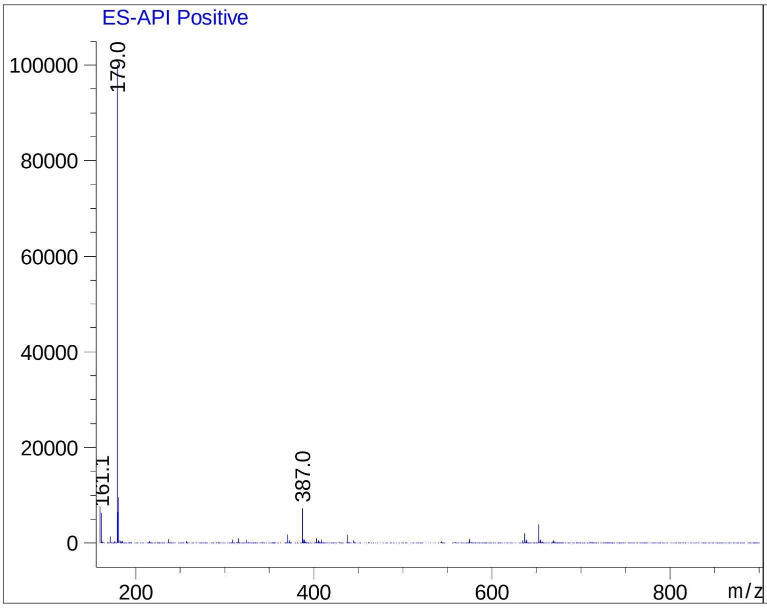


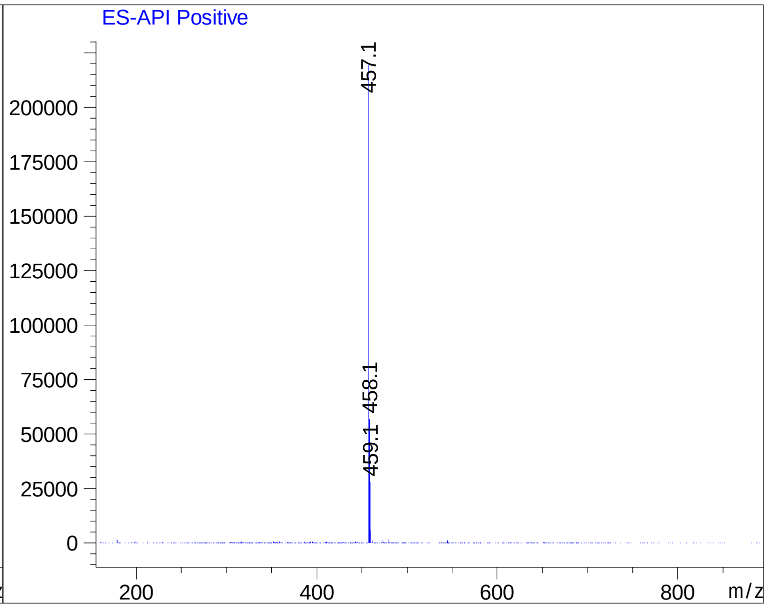

File ..mschem\04-23\200423-NCGC00264097-01-08266.D Tgt Mass (EZX): 495.08
Injection Date : 20-Apr-23, 12:19:39 Seq. Line : 0
Sample Name : NCGC00264097-01 Location : D2F-C1
Acq. Operator : Hsiu-Ling Lin Inj : 1
Spec. Reported : UV Integration Inj Volume : -3 ul
Acq. Method : C:\Users\Public\Documents\ChemStation\1\Methods\FINAL_GRAD_NO_PRINT.M
Analysis Method : C:\Users\Public\Documents\ChemStation\1\Methods\FINAL_GRAD_NO_PRINT.M
Sample Info : 0344122556 WalkUp method: 'FINAL_GRD_NO PRINT' Mol Wt: 495.08
Method Info : FINAL GRD BUT NO PRINT

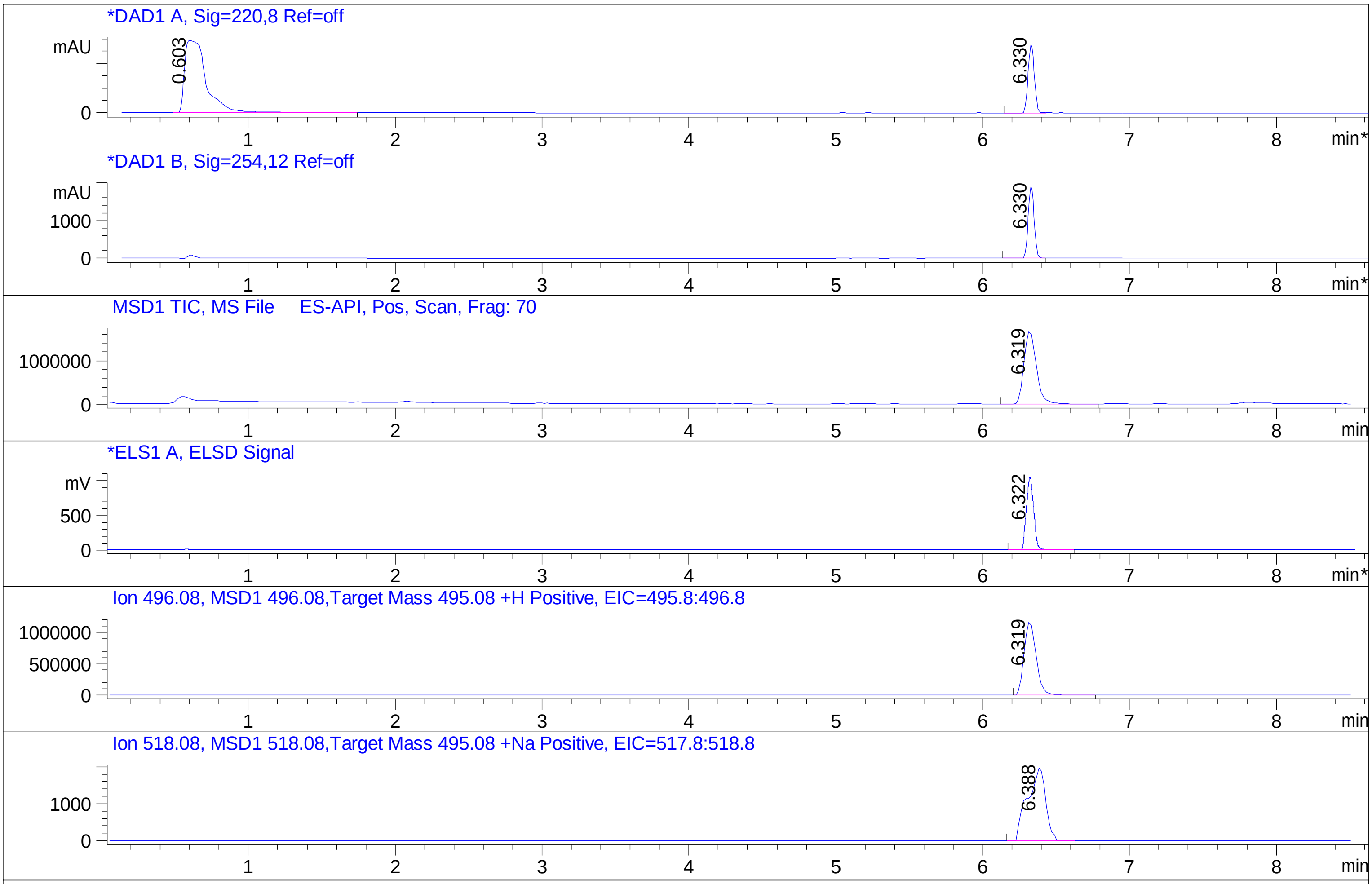


Integration Results for DAD1 A, Sig=220,8 Ref=off

| RetTim | Width | Area | Height | Area% | MS(+) |
|---|---|---|---|---|---|
| 0.60 | 0.14 | 30207.46 | 2931.89 | 78.21 | 179 |
| 6.33 | 0.05 | 8414.49 | 2798.97 | 21.79 | 496 |

Integration Results for DAD1 B, Sig=254,12 Ref=off

| RetTim | Width | Area | Height | Area% | MS(+) |
|---|---|---|---|---|---|
| 6.33 | 0.05 | 5461.47 | 1913.89 | 100.00 | 496 |

Ret. Time: 0.60  <<<< POSITIVE SPECTRA >>>>  Ret. Time: 6.33

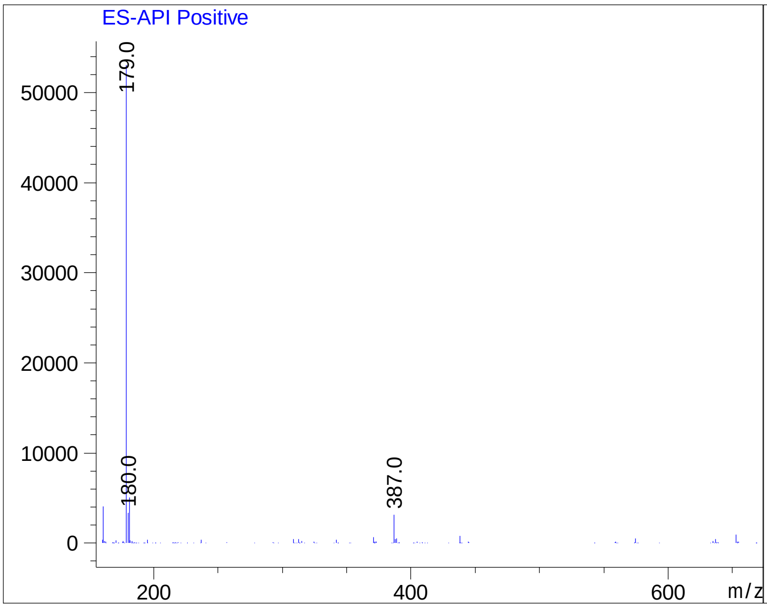


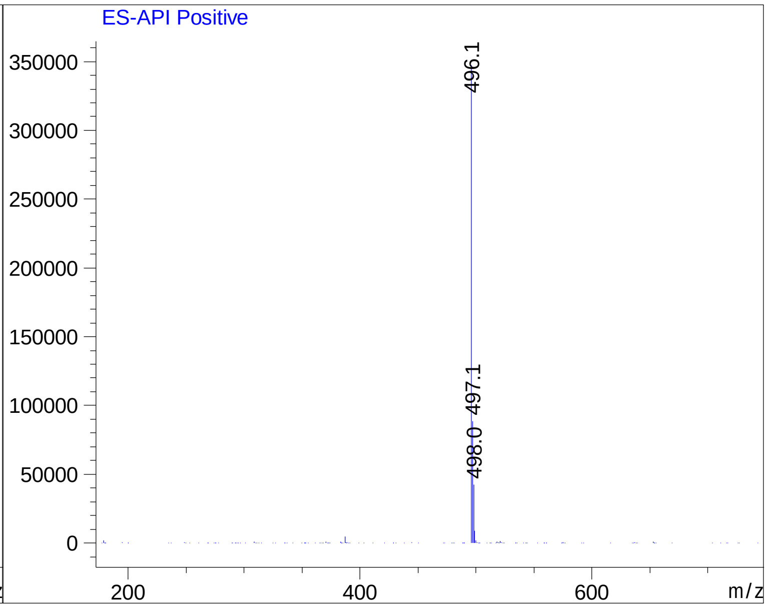

File ..mschem\03-23\310323-NCGC00371011-01-08130.D Tgt Mass (EZX): 366.2
Injection Date : 31-Mar-23, 15:42:57 Seq. Line : 0
Sample Name : NCGC00371011-01 Location : D1B-D5
Acq. Operator : Hsiu-Ling Lin Inj : 1
Spec. Reported : UV Integration Inj Volume : -3 ul
Acq. Method : C:\Users\Public\Documents\ChemStation\1\Methods\FINAL_GRAD_NO_PRINT.M
Analysis Method : C:\Users\Public\Documents\ChemStation\1\Methods\FINAL_GRAD_NO_PRINT.M
Sample Info : 0197505291 WalkUp method: 'FINAL_GRD_NO PRINT' Mol Wt: 366.2
Method Info : FINAL GRD BUT NO PRINT

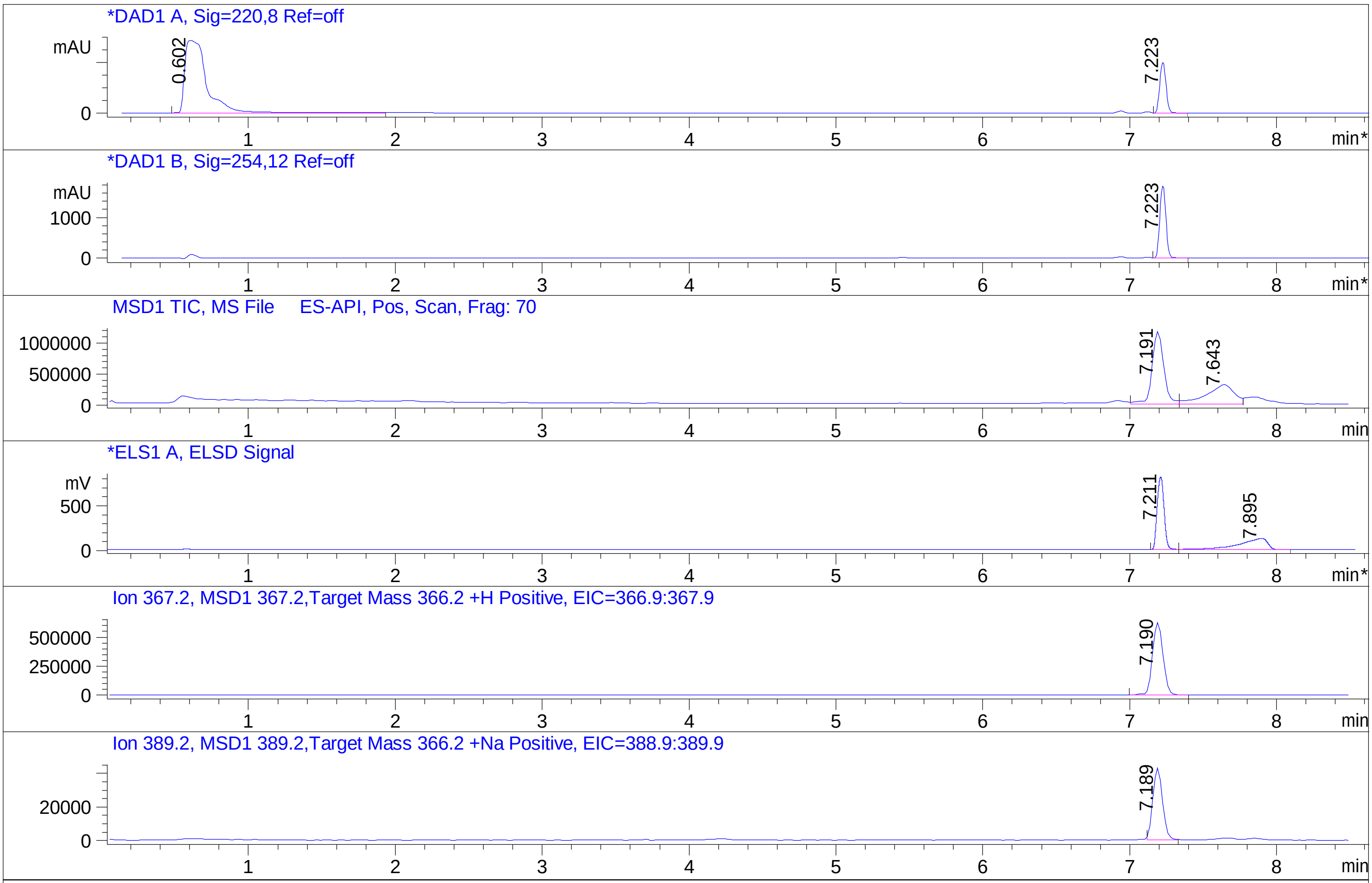


Integration Results for DAD1 A, Sig=220,8 Ref=off

| RetTim | Width | Area | Height | Area% | MS(+) |
|---|---|---|---|---|---|
| 0.60 | 0.16 | 30042.11 | 2863.28 | 83.44 | 179 |
| 7.22 | 0.05 | 5964.45 | 2007.13 | 16.56 | 367 |

Integration Results for DAD1 B, Sig=254,12 Ref=off

| RetTim | Width | Area | Height | Area% | MS(+) |
|---|---|---|---|---|---|
| 7.22 | 0.05 | 5128.50 | 1781.20 | 100.00 | 367 |

Ret. Time: 0.60  <<<< POSITIVE SPECTRA >>>>

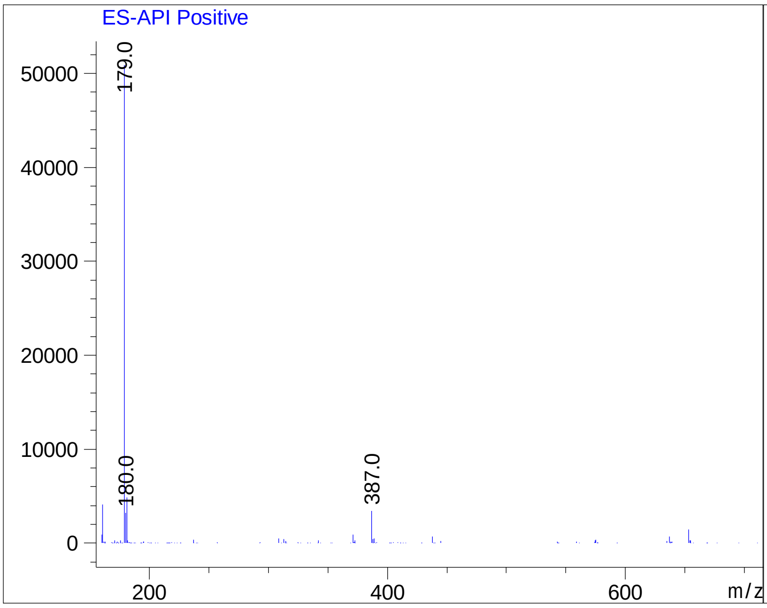


Ret. Time: 7.22

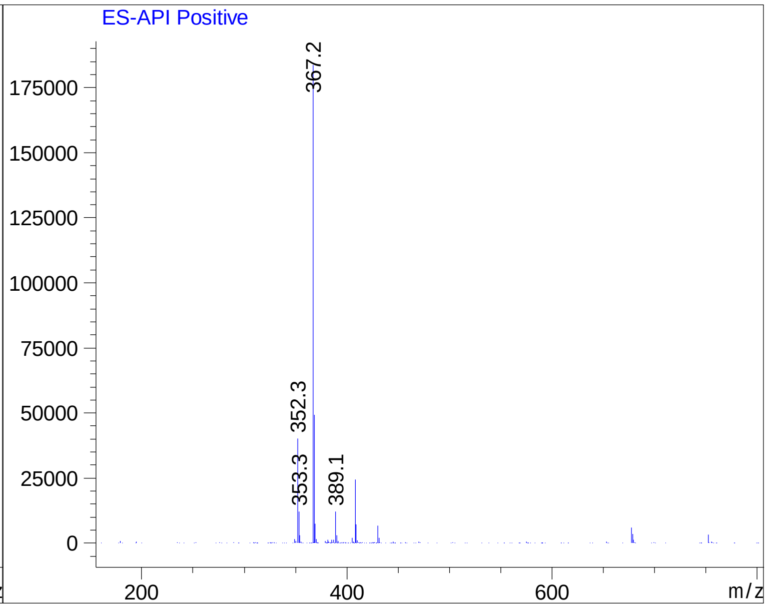

File ..mschem\04-23\200423-NCGC00378976-02-08264.D Tgt Mass (EZX): 615.15
Injection Date : 20-Apr-23, 11:59:44 Seq. Line : 0
Sample Name : NCGC00378976-02 Location : D2F-A1
Acq. Operator : Hsiu-Ling Lin Inj : 1
Spec. Reported : UV Integration Inj Volume : -3 ul
Acq. Method : C:\Users\Public\Documents\ChemStation\1\Methods\FINAL_GRAD_NO_PRINT.M
Analysis Method : C:\Users\Public\Documents\ChemStation\1\Methods\FINAL_GRAD_NO_PRINT.M
Sample Info : 0197502246 WalkUp method: 'FINAL_GRD_NO PRINT' Mol Wt: 615.15
Method Info : FINAL GRD BUT NO PRINT

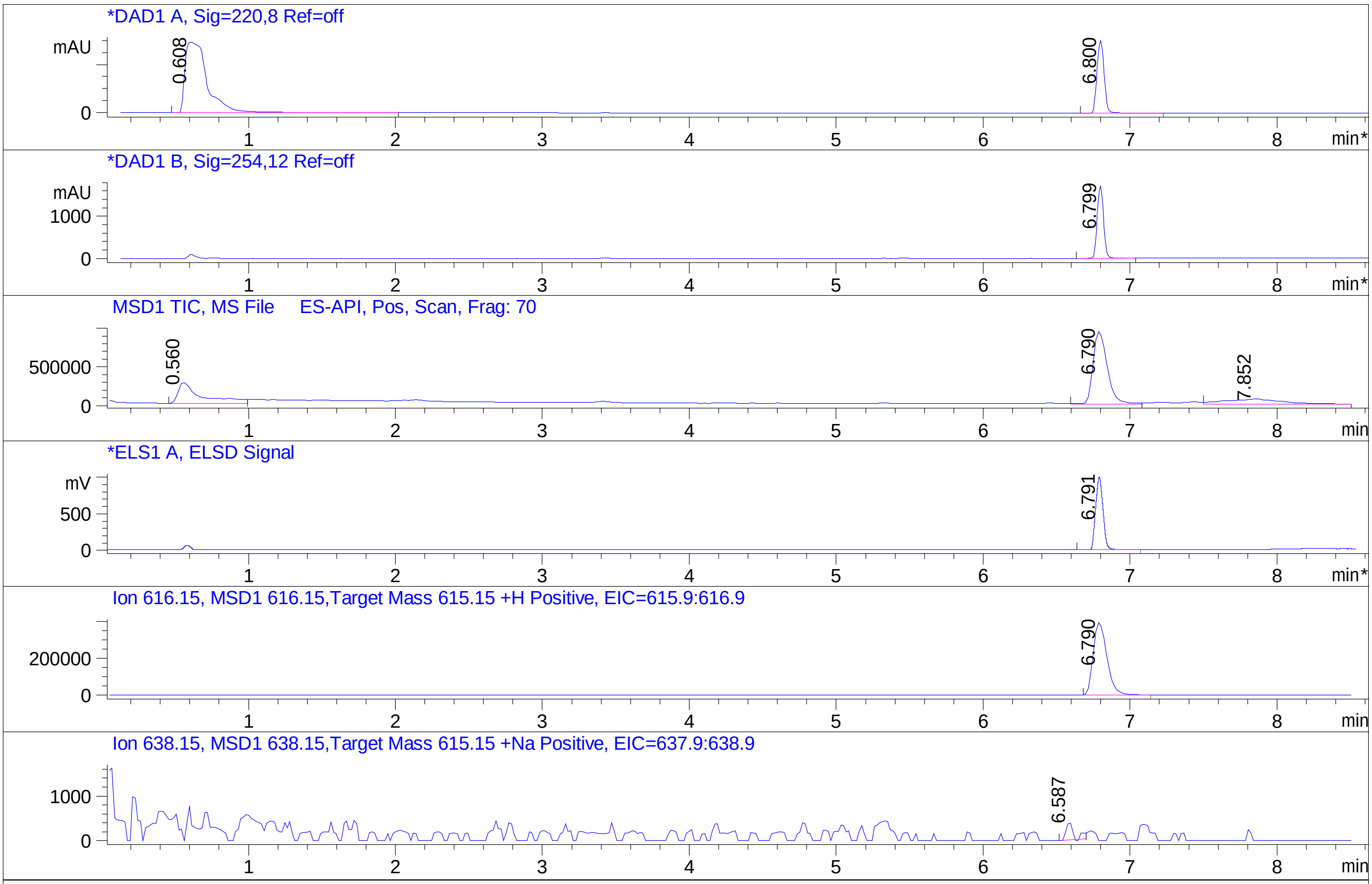


Integration Results for DAD1 A, Sig=220,8 Ref=off

| RetTim | Width | Area | Height | Area% | MS(+) |
|---|---|---|---|---|---|
| 0.61 | 0.14 | 31282.71 | 2930.15 | 75.35 | 179 |
| 6.80 | 0.06 | 10235.22 | 3021.27 | 24.65 | 616 |

Integration Results for DAD1 B, Sig=254,12 Ref=off

| RetTim | Width | Area | Height | Area% | MS(+) |
|---|---|---|---|---|---|
| 6.80 | 0.05 | 5157.51 | 1714.96 | 100.00 | 616 |

Ret. Time: 0.61 <<<< POSITIVE SPECTRA >>>> Ret. Time: 6.80

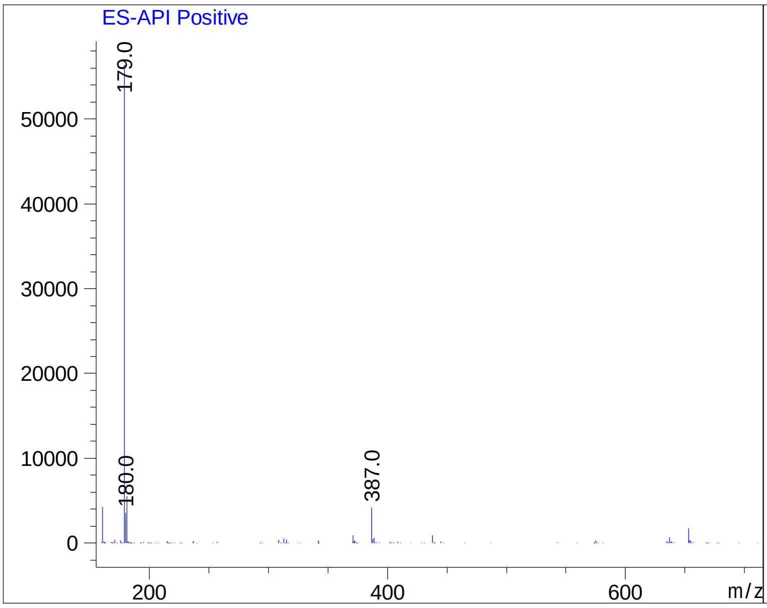


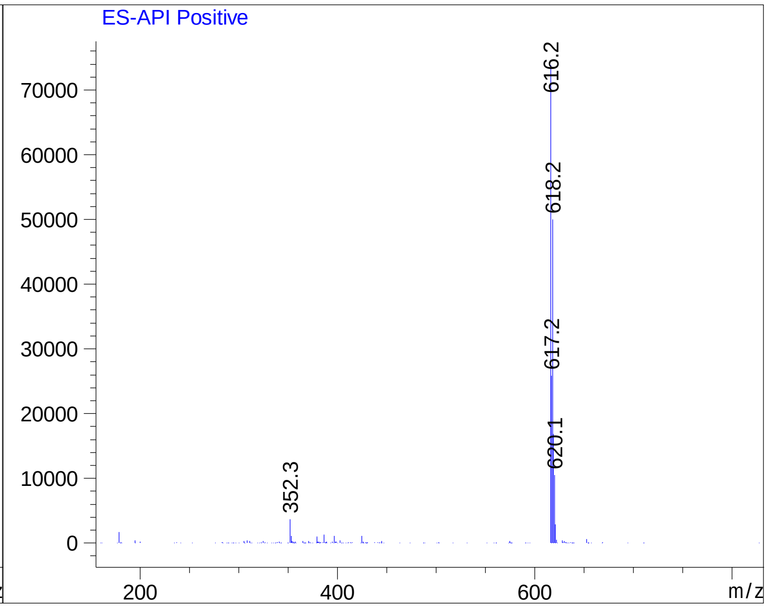

File ..mschem\04-23\200423-NCGC00386948-05-08265.D Tgt Mass (EZX): 438.14
Injection Date : 20-Apr-23, 12:09:42 Seq. Line : 0
Sample Name : NCGC00386948-05 Location : D2F-B1
Acq. Operator : Hsiu-Ling Lin Inj : 1
Spec. Reported : UV Integration Inj Volume : -3 ul
Acq. Method : C:\Users\Public\Documents\ChemStation\1\Methods\FINAL_GRAD_NO_PRINT.M
Analysis Method : C:\Users\Public\Documents\ChemStation\1\Methods\FINAL_GRAD_NO_PRINT.M
Sample Info : 0222217411 WalkUp method: 'FINAL_GRD_NO PRINT' Mol Wt: 438.14
Method Info : FINAL GRD BUT NO PRINT

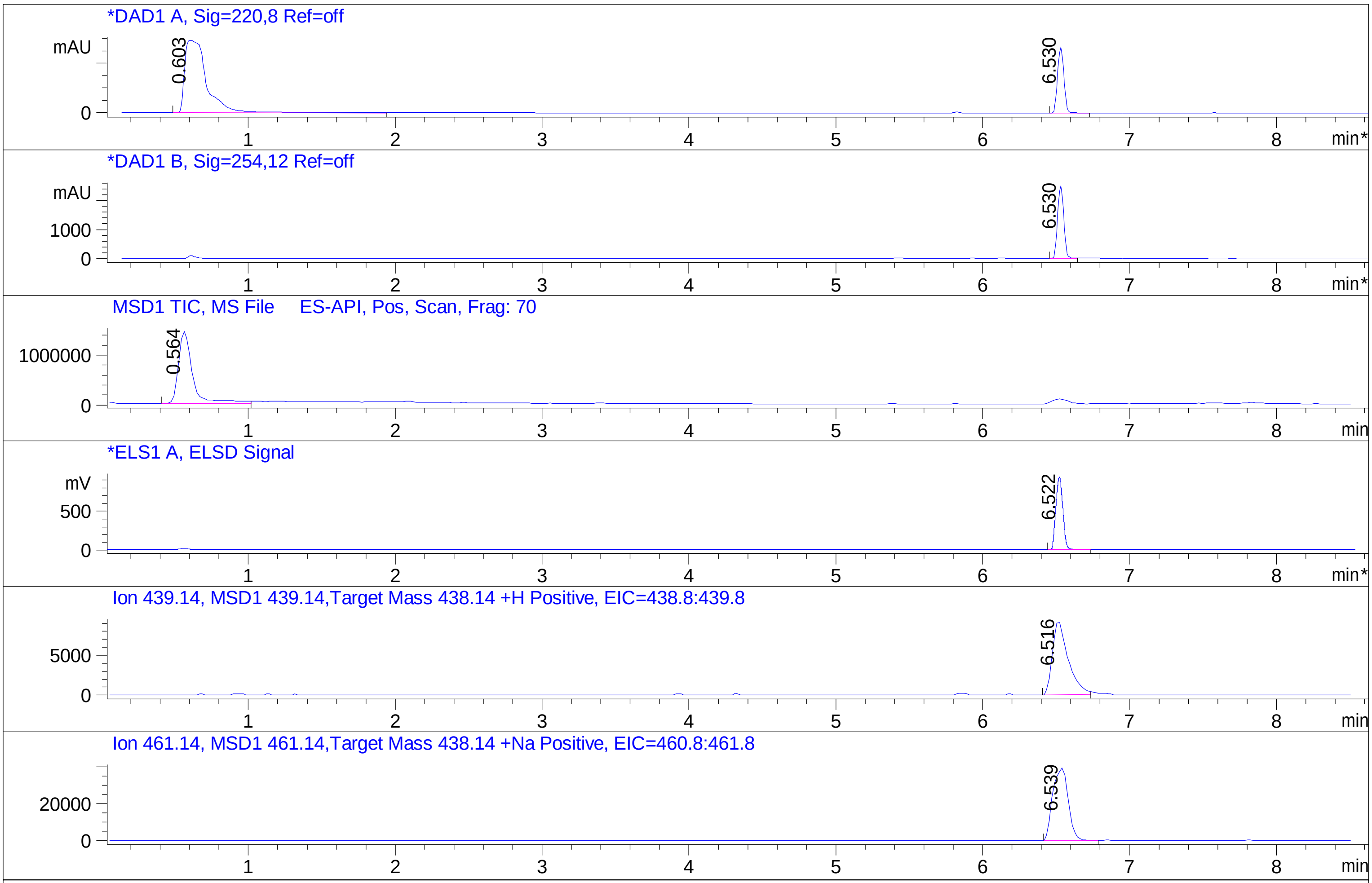


Integration Results for DAD1 A, Sig=220,8 Ref=off

| RetTim | Width | Area | Height | Area% | MS(+) |
|---|---|---|---|---|---|
| 0.60 | 0.14 | 30716.62 | 2913.66 | 79.22 | 179 |
| 6.53 | 0.05 | 8055.36 | 2649.42 | 20.78 | 461 |

Integration Results for DAD1 B, Sig=254,12 Ref=off

| RetTim | Width | Area | Height | Area% | MS(+) |
|---|---|---|---|---|---|
| 6.53 | 0.05 | 7356.02 | 2512.65 | 100.00 | 461 |

Ret. Time: 0.60  <<<< POSITIVE SPECTRA >>>>  Ret. Time: 6.53

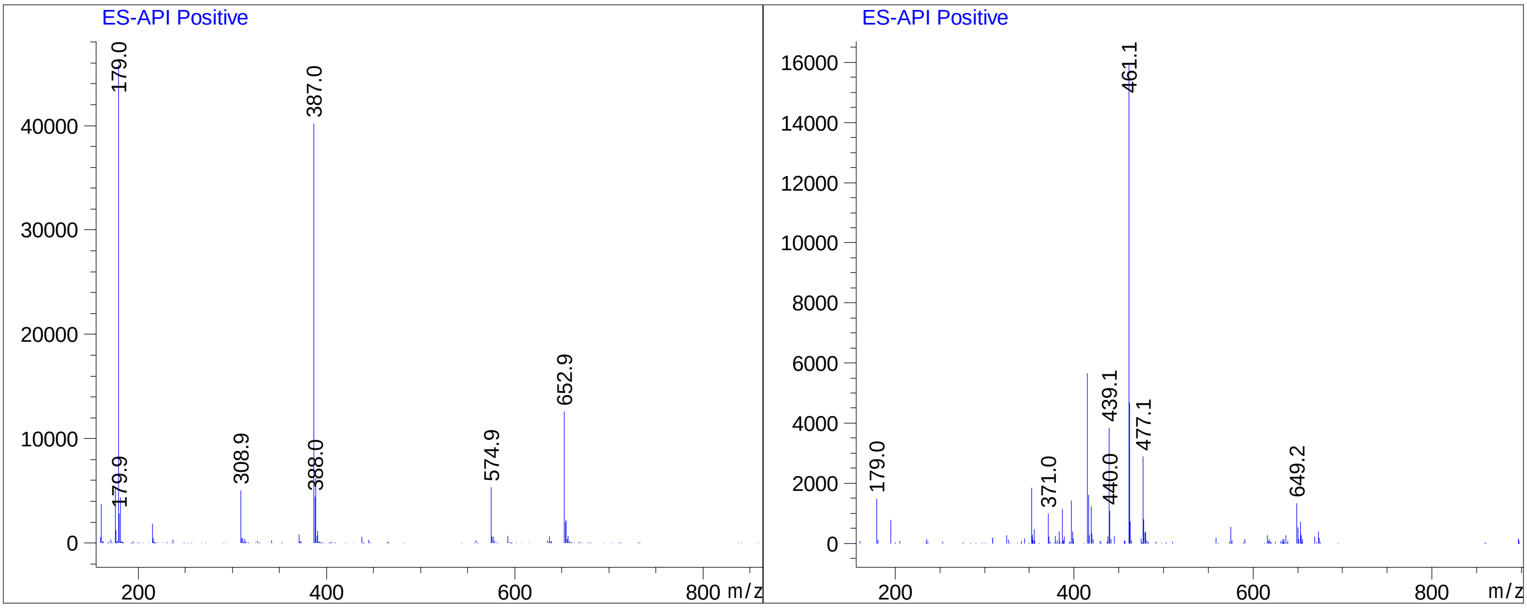

Supplement: Supplementary file 1 [file molecules-31-01043-s001.zip › molecules-4136545-supplementary.pdf]
